# Modularity and anti-modularity in networks with arbitrary degree distribution

**DOI:** 10.1186/1745-6150-5-32

**Published:** 2010-05-06

**Authors:** Arend Hintze, Christoph Adami

**Affiliations:** 1 Keck Graduate Institute of Applied Life Sciences, 535 Watson Drive, Claremont, CA 91711, USA

## Abstract

**Background:**

Much work in systems biology, but also in the analysis of social network and communication and transport infrastructure, involves an in-depth analysis of local and global properties of those networks, and how these properties relate to the function of the network within the integrated system. Most often, systematic controls for such networks are difficult to obtain, because the features of the network under study are thought to be germane to that function. In most such cases, a surrogate network that carries any or all of the features under consideration, while created artificially and in the absence of any selective pressure relating to the function of the network being studied, would be of considerable interest.

**Results:**

Here, we present an algorithmic model for growing networks with a broad range of biologically and technologically relevant degree distributions using only a small set of parameters. Specifying network connectivity via an assortativity matrix allows us to grow networks with arbitrary degree distributions and arbitrary modularity. We show that the degree distribution is controlled mainly by the ratio of node to edge addition probabilities, and the probability for node duplication. We compare topological and functional modularity measures, study their dependence on the number and strength of modules, and introduce the concept of anti-modularity: a property of networks in which nodes from one functional group preferentially do not attach to other nodes of that group. We also investigate global properties of networks as a function of the network's growth parameters, such as smallest path length, correlation coefficient, small-world-ness, and the nature of the percolation phase transition. We search the space of networks for those that are most like some well-known biological examples, and analyze the biological significance of the parameters that gave rise to them.

**Conclusions:**

Growing networks with specified characters (degree distribution and modularity) provides the opportunity to create surrogates for biological and technological networks, and to test hypotheses about the processes that gave rise to them. We find that many celebrated network properties may be a consequence of the way in which these networks grew, rather than a necessary consequence of how they work or function.

**Reviewers:**

This article was reviewed by Erik van Nimwegen, Teresa Przytycka (nominated by Claus Wilke), and Leonid Mirny. For the full reviews, please go to the Reviewer's Comments section.

## Background

The representation of complex interacting systems as networks has become commonplace in modern science [1-5]. While such a representation in terms of nodes and edges is near-universal, the systems so described are highly diverse. They range from biological (e.g., protein interaction graphs, metabolic reaction networks, neuronal connection maps) over engineering (blueprints, circuit diagrams, communication networks) to social systems (friends, collaboration, or citation networks). One of the hallmarks of human-designed systems appears to be their modularity [6]: systems designed in a modular fashion are more robust to component failure, can be quickly repaired by switching out defective modules, and their designs are easier to understand for a human engineer. Systems that emerged via biological evolution rather than design do not have to be easily understandable, but robustness and repair are still important characteristics. Beyond those, it appears that biological systems need to be *evolvable *[7-9]. While this criterion seems circular because obviously biological systems *have *evolved, there are differences in the degree of evolvability, which determine how well a system can adapt to changing environments. Modularity has been identified as possibly a key ingredient in evolvability, because it can both supply mutational robustness via the isolation of components and fast adaptation via the recombination of parts, or by altering the connections between the modules [8,10-13]. While our intuitive understanding of modularity is simple (from a designer's point of view) as "discrete entities whose function is separable from those of other modules" [10], the identification of modules from a representation of the system as a network is not straightforward. Commonly, modules in networks are identified via clustering algorithms that identify groups of strongly interconnected nodes that are only weakly connected to other such nodes [14-17], but often information external to the purely topological structure is used to determine modular relationships, such as co-regulation [18,19] or evolutionary conservation [20-22]. When the modular or community structure of a network is given or known, different measures exist to quantify the *extent *of modularity in the network [17,23-27].

Another defining characteristic of networks is their edge (or degree) distribution: the probability *p*(*k*) that a randomly chosen node of the network has *k *edges. Regular graphs, for example, are networks where each node has exactly the same number of edges as any other (a square lattice is a regular graph of degree four, except for the edge and corner nodes). Graphs can also be constructed randomly, by adding edges between nodes with a fixed probability. The first description of the connectivity distribution of such random graphs is due to Erdös and Rényi [28-30] and Solomonoff and Rapoport [31]. These authors found that the distribution of edges in such graphs is binomial, or, in the limit of a large number of nodes, approximately Poisson. While networks with such a degree distribution can be found in social interaction and engineering networks [32], they are comparatively rare in nature. For example, the edge distribution of the only biological neural network mapped to date (the brain of the nematode *C. elegans*) [33] is consistent with that of an Erdös-Rényi network [32,34].

Most other networks found in nature, however, have a *scale-free *edge distribution, implying that just a few nodes have very many edges, while most nodes are connected to only a few. The emergence of this scale-free degree distribution can be understood in many different ways [35-38] (see also [39,40]) and usually requires a *growth process *where either nodes with many edges preferentially attach to other nodes with many edges, or else grow via node duplication and mutation [41] (see [42] for a review of growth models). Indeed, graphs obtained by a growth process appear to show preferential attachment naturally [37] (because the oldest nodes usually have more edges than younger nodes) and are fundamentally different from those where edges are placed between nodes probabilistically [43]. Many other network's degree distributions are the result of a growth process even though the models currently in existence cannot produce them. The model we present here can be used to generate a wide variety of networks, and can be used to study a number of practical issues that arise when studying networks. For example, the model can falsify any hypothesis about network evolution that claims that a particular functional constraint is necessary for the evolution of a network feature, because no constraints other than the growth process and module connectivity restrictions are placed on the process. The purpose of this work is to produce a tool that allows a user to create networks with baseline characteristics to study network growth, and produce null models for the purpose of hypothesis testing. At the same time, the model can be used to create hypotheses about the processes that were in force historically when a network was formed, by finding the parameters that produce networks with similar structure.

For most of the applications studied here, we use a set of five independent probabilities to grow networks, which is not sufficient to produce networks with arbitrary degree distributions, but which appear to produce most of the biologically and technologically relevant degree distributions while generating many interesting distributions interpolating between them. In order to generate any *particular *degree distribution, the parameters in an assortativity matrix allow you to specify a network's ultimate connectivity directly. We use this matrix predominantly to generate graphs with defined functional modules, and study how modularity depends on a number of different parameters. We also introduce a new measure of functional modularity that only takes into account whether or not nodes that have been assigned to the same functional group connect to each other. Using this measure, we can show that some classes of networks can be *anti-modular*, that is, they show a tendency of nodes with the same functional assignment *not *to be connected to each other. Finally, we use the network growth model to investigate global properties of networks, and study the set of parameters giving rise to networks similar to well-known biological networks.

## Models and Methods

A fundamental difference between random graphs (defined as graphs obtained via a random process of edge addition resulting in degree distributions of the Erdös-Rényi type) and networks with scale-free edge distribution is thought to be the way in which these networks are generated. Scale-free networks are usually generated by growth via preferential attachment [35,43-46] or else grown via duplication with subsequent diversification [41,47,48]. For some networks (mostly metabolic reaction networks [49,50]) preferential attachment is not sufficient to explain their degree distribution [51]. Here we describe an algorithm that will grow networks with a broad range of degree distributions based on a growth model with only a few parameters. Depending on those parameters, we can obtain Erdös-Rényi-like graphs, networks with scale-free degree distribution, small-world networks, regular graphs and lattices, bi-partite and *k*-partite graphs, and anything in between. In addition, this algorithm is able to grow those networks with any degree of modularity and arbitrary size. The growth parameters can even be chosen in such a way that the resulting networks actually show a negative modularity score, that is, they can be *anti-modular*.

**Figure 1:**
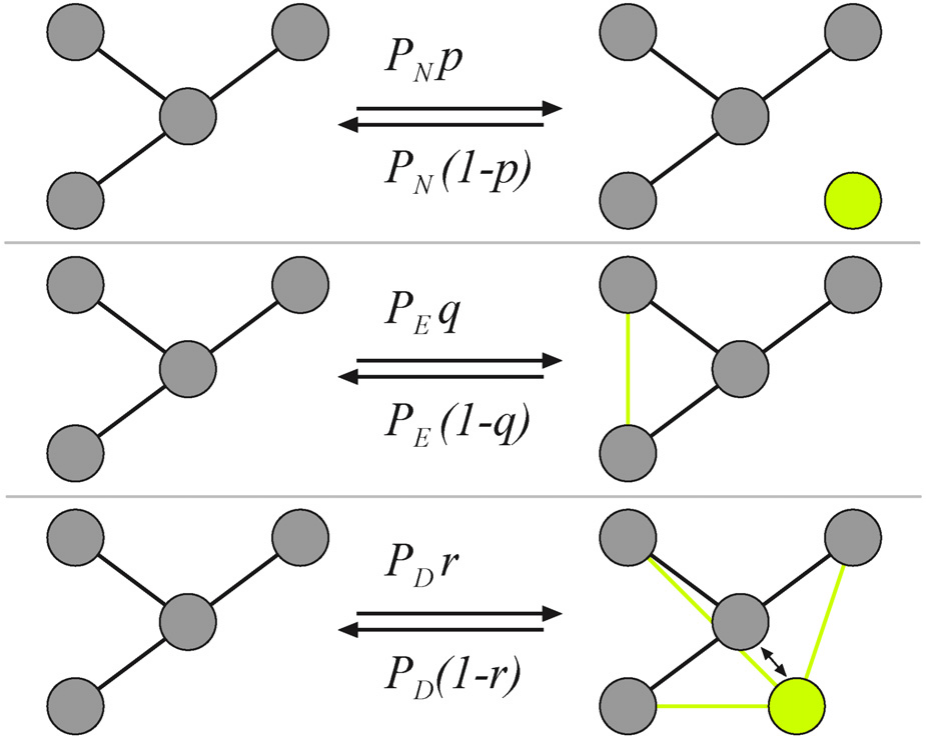
**Network growth events and probabilities**. Growth (or shrinkage) of network is determined by node, edge, and duplication events with specified probabilities, as described in the text.

Our networks are usually generated from a single seed node, but can take any specified initial configuration of nodes and edges as starting condition. Subsequent *events*, determined by user-chosen probabilities, occur stochastically and usually lead to network growth. For example, the *node-event probability P*_*N *_determines that a node is either added (without edges) or deleted, depending on a second parameter, the *node addition *probability *p*. Thus, for a single node event, the probability that a node will be added is *pP*_*N *_while the probability that a node will be deleted is *P*_*N *_(1-*p*) (see Figure 1). A second type of event affects edges with probability *P*_*E*_: the *edge-event probability*. Just as with nodes, edges will be added with an *edge-addition probability q*, so that a single edge event will add an edge with probability *qP*_*E *_while an edge is removed with probability (1-*q*)*P*_*E*_. Note that while node addition or removal happens unconditionally, edge addition or removal is not guaranteed. The algorithm will only place an edge if the pair of nodes that is randomly selected is unconnected. Similarly, an edge removal instruction is only carried out if the pair of nodes that is randomly selected already has a connection, and otherwise fails. As a consequence, even edge addition probabilities *q *< 0.5 will lead to a steady-state distribution of edges to nodes. The main parameters of the growth model are summarized in Table 1. Note that because a rescaling of the probabilities *pP*_*N*_, *qP*_*E*_, and *rP*_*D *_by a common factor only changes the time it takes to achieve a network of a particular size, these probabilities are not independent (for example, *pP*_*N *_can be used to rescale all parameters). However, for many applications it is interesting to vary these probabilities independently.

**Table 1:** Parameters of network growth model

Abbreviation	probability
*P*_*N*_	Node-event probability
*P*_*E*_	Edge-event probability
*P*_*D*_	Duplication-event probability (duplication or fusion)
*p*	Conditional node-addition probability (given a node event). Conditional probability of node removal is 1 - *p*
*q*	Conditional edge-addition probability (given an edge event). 1 - *q *is the conditional probability that an edge is removed
*r*	Conditional node-duplication probability (given a duplication/fusion event). Conditional node-merging probability is 1 - *r*

We can calculate the steady-state distribution of edges per node (mean degree ⟨*k*⟩) by calculating the total number of nodes *n *and edges *m *as a function of the number of events *N *and the parameters *P*_*N*_, *P*_*E*_, *p*, and *q *(we do not consider duplications in this calculation). The number of nodes added per event is *pP*_*N *_and the number of nodes removed is (1- *p*)*P*_*N*_, so that the net number of nodes added after *N *events(1)

The net number of edges *m *added is more complicated, because edges are only added with probability *q*(1 - *ξ*), and removed with probability (1 - *q*)*ξ *per event, where *ξ *is the graph sparseness , and represents the probability that a random pair of nodes is connected by an edge. At the same time, every time a node is removed, the algorithm removes the edges attached to it. On average then, a node removal event subtracts the average degree of that node, which is ∑_*i*_*k*_*i*_/*n *= 2*m/n *= ⟨*k*⟩, so that(2)

We can then write an equation for the asymptotic dependence of the mean degree(3)

or(4)

where *η *= *P*_*E*_/*P*_*N*_, and using *ξ *≈ ⟨*k*⟩/*n*, which holds for large *n*. Thus we see that in the limit of large *n*,(5)

a behavior that is borne out in the simulations (data not shown).

A third type of event leads to node duplication or merger (fusion), controlled by the parameter *P*_*D*_. Here, a node is duplicated with probability *rP*_*D*_, while two nodes are fused with probability (1 - *r*)*P*_*D *_(the parameters are summarized in Table 1). While edge and node events are straightforward, node duplication/fusion events need more explanation because there are several different ways in which nodes can be duplicated or fused. Here, we implement an algorithm in which node duplication is directly related to the concept of modules: When duplicating a node, the new node is by definition in the *same module *as its ancestor, and the new node is connected to all nodes the ancestor is connected to. In order to implement this, nodes have to be assigned a *tag *that determines the module they belong to, the moment the node is created. (It is convenient to represent different tags by different *colors*, so we often refer to nodes in different modules-that is, carrying different tags-as having different colors).

In order to assign a module to a duplicated node, the number of modules has to be given at the outset, and the probability for a node of color *k *to connect to a node of color ℓ is obtained from the assortativity matrix *e*, which stores the fraction of edges between pairs of colors. For *N*_*c *_modules *M*_1_,...,, this matrix can be written as(6)

For the case of node merging, two nodes (A and B) are picked at random. Node A keeps its connections and in addition obtains all the connections that node B had, upon which node B is deleted. The selection of the nodes to be merged is module independent, and thus could either merge nodes within a module or across modules.

When growing modular networks, nodes are assigned a color based on a vector of probabilities that can be specified beforehand (for all the results in this paper, nodes are assigned to a module randomly at the time they are created). If an edge event specifies the placement of an edge, a random pair of nodes is selected and the identity of the colors determined. At this time, a random uniform number is drawn, and the edge is placed if this number is smaller than the corresponding probability in the *e*-matrix (6). If no edge is set, the algorithm tries to set the edge for a different pair of nodes, and attempts this up to 1,000 times.

We determine a growth stop criterion either by specifying a maximum number of nodes, or by specifying a fixed number of iterations of the algorithm. In principle, the algorithm allows for the generation of both directed and undirected graphs. Here, we restrict ourselves to networks with undirected edges, and furthermore prevent nodes from connecting to themselves. Finally, two different growth initial conditions are possible: one in which we start with a single node from the first module in the *e*-matrix, the other where we start with a single node from every module. The *N*_*c*_(*N*_*c *_- 1)/2 entries in the assortativity matrix, together with the number of colors and the six probabilities for stochastic network growth described above fully determine the structure of the network.

### Software availability

The program to grow the networks described in this article will be made freely available.

## Results and Discussion

### Growing networks with complex degree distributions

The standard model for growing graphs with exponential degree distribution is due to Callaway et al. [43], who introduced a model where a node is added at each event, and an edge is added with a given probability per event. While there are no duplications in this model and edges are not preferentially attached to high-degree nodes, there is still a form of preferential attachment because older nodes have had more opportunities of obtaining edges, and also have a higher probability of connecting to nodes with more edges [43]. This model produces exponential degree distributions whose form can be predicted exactly, but scale-free distributions cannot be produced. With the present network growth model, it is easy to grow networks with more complex degree distribution, by changing just a few parameters.

**Figure 2:**
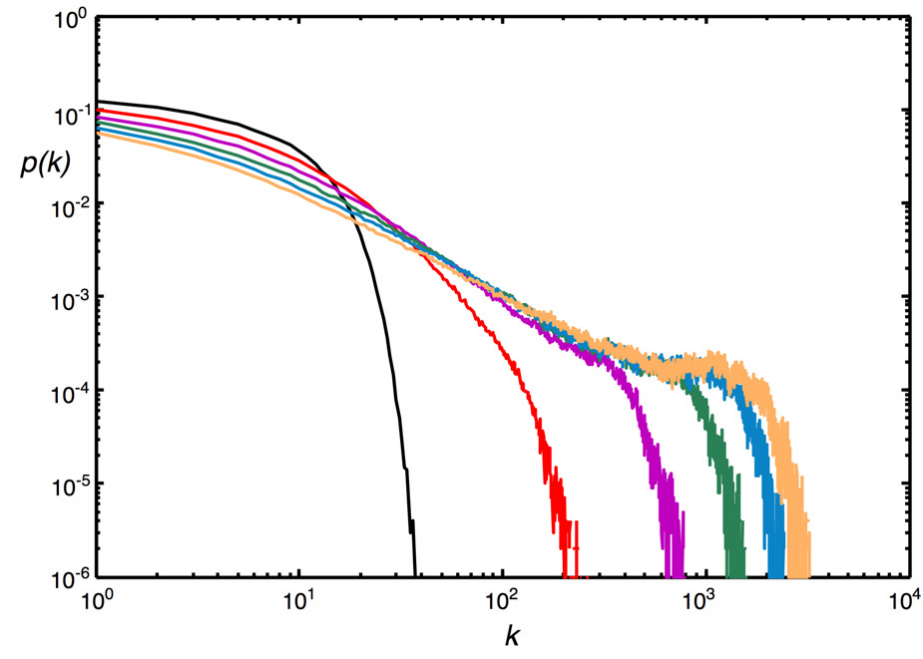
**Degree distribution as a function of duplications**. The degree distribution of randomly grown networks with different node duplication probabilities *P*_*D *_(*r *= 1), at fixed *P*_*N *_= 0.2, *P*_*E *_= 0.75 (with *p *= 1, *q *= 1). *P*_*D *_= 0 (black), *P*_*D *_= 0.1 (red), *P*_*D *_= 0.2 (magenta), *P*_*D *_= 0.3 (green), *P*_*D *_= 0.4 (blue), and *P*_*D *_= 0.5 (yellow). Average of 100 replicates of networks grown to size *n *= 1,000.

**Figure 3:**
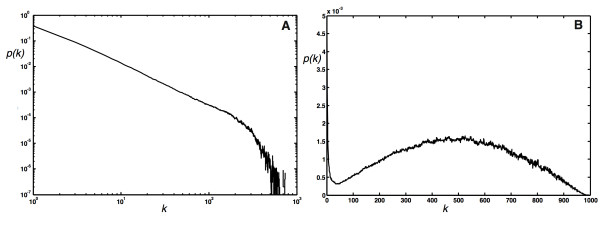
**Edge distribution of networks grown with different parameters**. (A) Scale-free edge distribution *p*(*k*) of networks obtained with a growth algorithm using *P*_*N *_= 0.2625, *p *= 1.0, *P*_*E *_= 0.15, *q *= 1.0, *P*_*D *_= 0.225, and *r *= 1.0, undirected edges, no modules, average over 1,000 networks grown to 10, 000 nodes. (B) Edge distribution of networks grown with *P*_*N *_= 0.002625, all other parameters as in (A), averaged over 10,000 networks grown to 1,000 nodes.

To begin with, we test whether the exponential distribution of the Callaway model morphs into a scale-free distribution as the duplication event probability is increased. In Fig. 2 we show the degree distribution with a fixed node and edge event probability, but changing the node duplication probability *P*_*D*_, and confirm that if networks grow with duplication, the scale-free edge distribution is unavoidable [41]. Networks that are scale-free over more decades actually emerge if nodes are added more often than edges (see Fig. 3A). Choosing a low *P*_*N*_, on the other hand, leads to the growth of networks with a Poisson-like degree distribution (Fig. 3B). Note that the scale-free and the Erdös-Rényi-type distributions depicted in Fig. 3 were obtained using the same set of parameters except that the node addition probability was 100 times less for the graph that resulted in an Erdös-Rényi-like edge distribution. In principle, keeping the relative ratio of the three probabilities *P*_*N*_, *P*_*E*_, and *P*_*D *_the same (when *p *= *q *= *r *= 1) results in the same edge distribution (see **Models and Methods**).

**Figure 4:**
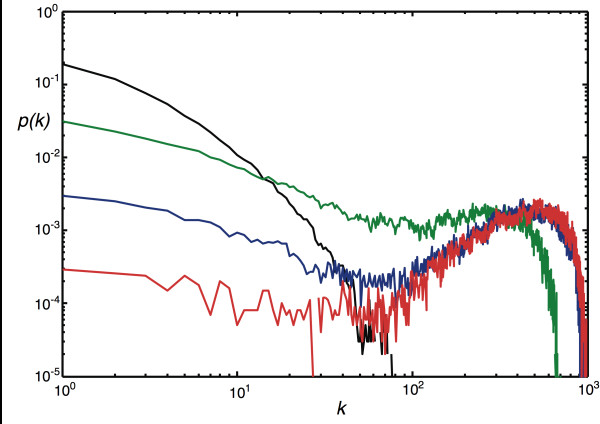
**Edge distributions for networks grown under different regimes**. *P*_*N *_= 1.0 (black line, exponential distribution) *P*_*N *_= 0.1 (green), *P*_*N *_= 0.01 (blue), and *P*_*N *_= 0.001 (red). All other parameters are set to *P*_*E *_= *p *= *q *= 0.75, *P*_*D *_= 0.5, *r *= 1.0. Networks are unmodular and undirected, grown to size *n *= 10, 000, averaged over 100 replicates.

**Figure 5:**
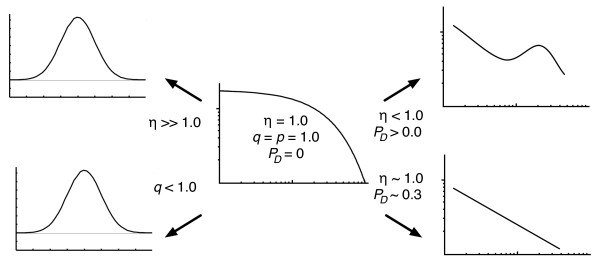
**Changing a network's degree distribution**. This sketch indicates how different degree distributions morph into one another as several different parameters of the growth model are changed. From the "default" exponential degree distribution of the Callaway model (center of diagram) Erdös-Rényi-like distributions can be obtained in two different ways: either by increasing the probability to add edges while keeping the node addition probability constant, or by randomizing edges using a small *q *(distributions on the left, note the non-logarithmic axes). Approximately scale-free distributions can be obtained from the exponential one by increasing the node duplication probability, but doing so while decreasing the number of edges per node creates a hybrid between scale-free and Erdös-Rényi-type distributions (distributions on the right).

Choosing probabilities in between the parameter values described allows us to grow very different networks, with edge distributions in between exponential and Poisson. For example, there are interesting "transition stages" where parameter combinations lead to networks that are neither scale-free nor Erdös-Rényi. We show in Fig. 4 extreme and intermediate edge distributions where we varied the node-event probability (*P*_*N*_) from 0.001 to 1.0 while keeping all other probabilities constant. The distribution obtained for *P*_*N *_= 0.001 has all the characteristics of an Erdös-Rényi-type edge distribution, such as the one depicted in Fig. 2B (note the difference in scales). We conclude that the edge distribution can be controlled entirely with the node addition probability and the edge duplication probability (as long as the edge addition probability is not too low): for low edge duplication, tuning *P*_*N *_from 1 to small values morphs the degree distribution from exponential to Erdös-Rényi. If the edge duplication probability is substantial, however, the same change in *P*_*N *_moves the distribution from scale-free to Erdös-Rényi. As a corollary, moving *P*_*D *_from small values to larger values for moderate to high *P*_*N *_changes an exponential towards a scale-free distribution, as we saw in Fig. 2. While we have not conducted an exhaustive parameter exploration, we can summarize how the main parameters affect the degree distribution in a qualitative manner. In the absence of duplication, the degree distribution is exponential or approximately Poisson, depending on the size of the ratio of the edge- to node-event probability *η *= *P*_*E*_/*P*_*N *_and the edge addition probability *P*_*D*_. For *q *= 1, the Callaway model [43] predicts an exponential edge probability distribution(7)

with mean number of edges per node (degree) ⟨*k*⟩ = 2*η *in agreement with Eq. (4) (see Fig. 5 center). If *η *becomes large, however, this distribution starts to resemble a Poisson distribution, as indicated in Fig. 5. An Erdös-Rényi-type distribution can also be obtained without touching *η*, by simply decreasing *q *(the probability that a node is added per edge event) because as long as edges are added slowly, a small enough *q *will lead to the random rewiring of a graph (see Fig. 5 lower left). In both cases (*η *≫ 1 or *q *< 1 while *η *≈ 1) the edge probability distribution quickly becomes independent of the network size. As we increase the node duplication probability, we move towards the distributions on the right hand size of Fig. 5. While the distribution starts to develop a form reminiscent of a power-law for low degrees when *η *< 1, the duplications lead to a hump at larger degrees. Whether or not this distribution is independent of the size of the network is unclear: when duplications enter the generation process, may of the graph properties depend not only on the initial configuration used for the graph, but also the length of the process. Likewise, it is unclear whether the parameters that give rise to power law distributions for a finite process (as in the lower right graph in Fig. 5) are the same if the size of the network tends to infinity. Fortunately, real-world networks are not infinite, so the model is useful for the generation of surrogate networks even if the asymptotic distribution is not known.

**Figure 6:**
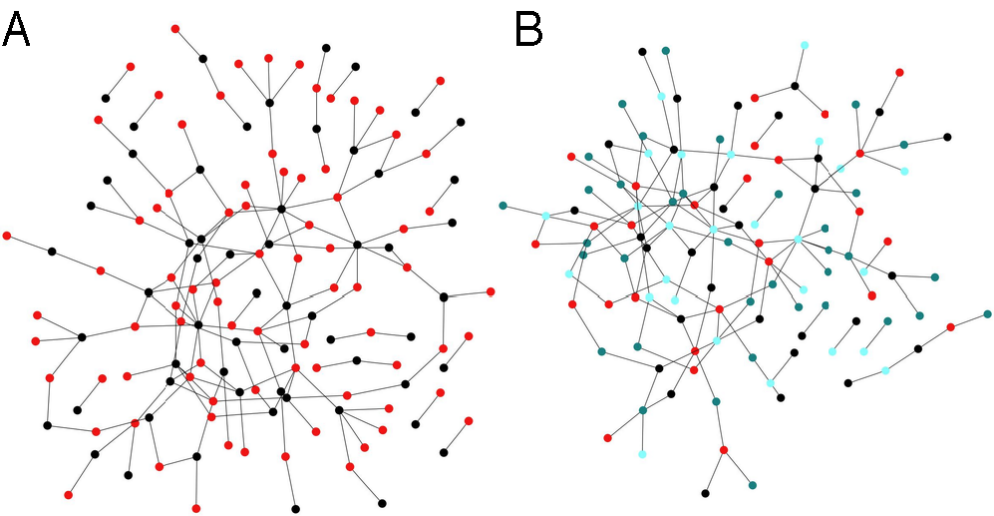
**Bipartite and *k*-partite graphs**. (A) Bipartite graph, nodes from one group colored in red, nodes from the other group colored in black, *P*_*N*_/*P*_*E *_= 0.3, *p *= 0.85, *q *= 0.75, *P*_*D *_= 0.0. The graph was grown for 1,000 iterations of the algorithm, with undirected edges. (B) Graph grown with the same parameters as (A), but for *k *= 4.

The algorithm can be used to create lattices with an arbitrary degree or connectivity by making use of the assortativity matrix in an unconventional manner: Each node of the lattice is assigned a unique module, where the probability of having an edge between modules reflects the desired neighborhood relations in the lattice. Instead of seeding the growth process with a single node, the algorithm is started with a fixed number of nodes and no edges, and *P*_*N *_= 0. The growth process then enacts a percolation problem with edge probability *qP*_*E*_, and a geometry dictated by the assortativity matrix. To create bipartite graphs with edges only connecting nodes from different groups, we can grow networks from an assortativity matrix with a vanishing diagonal [see Fig. 6A]. Nearly bipartite graphs are obtained by varying the entries in the matrix accordingly. Clearly, the algorithm can generate arbitrary *k*-partite graphs, by extending the dimension of the assortativity matrix. We show in Figure 6B a network iterated for 1,000 steps with the same parameters as Fig. 6A, but with *k *= 4 (nodes colored according to the group label).

### Modularity

That biological, technological, and social networks are organized in a modular fashion is by now a commonplace observation. Yet, there is no standard measure of modularity, nor is there a standard algorithm that will partition networks into modules. There are several reasons for this apparent shortcoming. On the one hand, while the term "modular organization" is fairly intuitive, anyone who is familiar with the structure of real-world networks understands that this intuitive notion can only be applied approximately, and with a good amount of prudence. Modules are often identified using the topological structure of the network, for example by counting the number of shortest paths between nodes, or by identifying an excess number of edges between nodes as compared to a random (Erdös-Rényi-type) network. However, it is also possible that groups of nodes function together as a module without any obvious topological signature. Furthermore, functional modules often overlap, while topological modules are usually defined in such a way that they are mutually exclusive. Therefore, we expect that topological and functional measures of network modularity can disagree, and that this disagreement can be more or less severe depending on the type of network under consideration.

We would also like to highlight the difference between modularity *measures*, which quantify the modularity in a network whose modules have already been determined, and *module-discovery *algorithms, which partition a network into groups of nodes. Often, module-discovery is performed by attempting to maximize a modularity measure, but in principle neither does a modularity measure imply an algorithm for module discovery, nor does a module-discovery algorithm necessitate a measure of modularity.

A commonly used measure of modularity is due to Newman [17], who assumes that modularity implies that nodes in the same module have more connections between them than would be expected for a random control, that is, a network where all the module assignments have been randomized. If *k*_*i *_is the number of edges of node *i*, and *m *= 1/2∑_*i *_*k*_*i *_is the total number of edges in the network, then the probability that two nodes *i *and *j *are connected by chance is *k*_*i*_*k*_*j*_/2*m *(as long as the degrees for node *i *and *j *are independent). Now, define the network adjacency matrix *A*, in such a way that *A*_*ij *_= 1 if node *i *connects to node *j *and *A*_*ij *_= 0 otherwise. This matrix is symmetric for undirected networks, and can have non-integer entries if the *strength *of a connection is taken into account. Here, we limit ourselves to undirected networks that have "binary" edges, but the extension is obvious. We furthermore limit ourselves to networks without node self-connections, which implies that the diagonal of *A *vanishes. If furthermore the module *assignment *for each node is known, we can define a modularity matrix *S *in such a way that *S*_*ij *_= 1 if nodes *i *and *j *belong to the same module, and zero otherwise. Newman's modularity *Q*_*N *_is then defined as [17](8)

There is clearly a certain amount of arbitrariness in modularity measures of this kind. For example, a different measure using similar ideas is often called the "assortativity" of a network. This measure is also due to Newman [25], and quantifies how likely it is that nodes of the same "kind" attach to each other, where "kind" can be any tag that is attached to a node to distinguish it from another class of nodes. As before, we refer to this tag as the node's color, so that assortativity measures how often nodes of the same color connect to each other rather than to nodes of a different color. Let us define the assortativity matrix *e *(sometimes called the "mixing matrix") such that *e*_*k*ℓ _gives the fraction of edges that attach a node of color *k *to a node of color ℓ, and *a*_*k *_= ∑_ℓ _*e*_*kl *_is the fraction of edges that either begin or end at a node of color *k *(we again restrict ourselves to undirected networks here, so that *e *is symmetric). Newman's assortativity is then given by [25](9)

Both measures (8) and (9) are bounded from above by 1, and they can both become negative (indeed, Newman's modularity and assortativity measures are closely related, see Appendix). While the assortativity is constructed in such a way that networks with random assignment of colors (modules) to nodes gives rise to a vanishing measure, this is not generally true for *Q*_*N*_. Furthermore, both measures can in principle detect in networks a tendency of nodes of the same module or color *not *to connect to each other (a phenomenon we call *anti-modularity *or *anti-assortativity*). However, the measures do not treat anti-modularity (or anti-assortativity) on the same footing as modularity or assortativity.

It is possible to introduce a measure of modularity that is closely related to both of Newman's measures, but gives more weight to "like"-edges if the number of colors is large. This is obtained by modifying the modularity matrix that enters Eq. (8) so that(10)

where *N*_*c *_is the number of modules or colors. (As in the following we will tag nodes that belong to the same *functional *module with the same color, we often refer to colors or modules interchangeably.) With such a modularity matrix, connections between nodes of unlike color are penalized, most heavily so if there are only a few colors. We define our functional modularity measure in terms of this generalized modularity matrix(11)

but note that we omitted the term -*k*_*i*_*k*_*j*_/2*m *in Newman's measure that subtracts the probability that two nodes connect at random. Indeed, the latter bias is typical for modularity measures that attempt to capture the way modules are reflected in network topology, while our measure *Q*_*H *_focuses on function only. Because *Q*_*H *_can also be written as (see Appendix)(12)

we see that *Q*_*H *_vanishes for non-associative (non-modular) networks, because when color is assigned randomly to nodes we have  so that Tr *e *= 1/*N*_*c*_. At the same time, *Q*_*H *_is maximal for graphs if colors only connect to like colors (Tr *e *= 1). But in contrast to Newman's measures, *Q*_*H *_can become significantly negative, more so if the number of modules is small. For bipartite graphs, for example (graphs with nodes of two colors where only unlike colors connect) we find Tr *e *= 0, so that *Q*_*H *_= -1.

**Figure 7:**
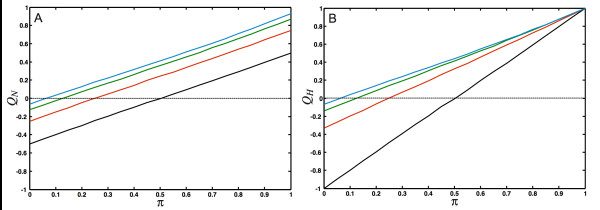
**Comparison of modularity metrics**. Comparison of the modularity measures defined in Eqs. (8) and (11). Networks with between 2 and 16 modules were grown depending on the intra-module edge probability *π*. (For *π *= 0 the networks are as anti-modular as possible and become *k*-partite (where *k *is the number of modules), while for *π *= 1 they are as modular as possible). (A): *Q*_*N *_[defined in Eq. (8)] for *N*_*c *_= 2 (2 modules, black line), *N*_*c *_= 4 (red), *N*_*c *_= 8 (green), *N*_*c *_= 16 (blue). (B) *Q*_*H *_[defined in Eq. (11)]. Colors as in (A). Each point was averaged over 50 networks with 1,000 nodes. The networks were grown with *P*_*N *_= 0.5, *p *= 1.0, *P*_*E *_= 1.0, *q *= 1.0 and *P*_*D *_= 0.0, using undirected edges.

To study how the different modularity measures depend on the number of modules in the network as well as the strength of the module's interconnectivity, we generate networks with a tunable amount of modularity. A simple model for generating modular networks is an assortativity matrix for *N*_*c *_colors where like-colors connect to each other with probability *π *(the intra-module edge probability), and connect to nodes of a different color with probability (1 - *π*)/(*N*_*c *_- 1), irrespective of color (the "equal opportunity" model, see Appendix). The probability 1 - *π *can then be viewed as an *inter-module *edge probability and can be used to dial between perfectly modular (*π *= 1) and perfectly anti-modular (*π *= 0) networks. The functional modularity *Q*_*H *_seen in Fig. 7B depends strongly on the number of modules, and is larger than *Q*_*N *_(depicted in Fig. 7A) for modular networks, and smaller than *Q*_*N *_for the anti-modular ones. Indeed, the inherent bias in Newman's measure for modules whose member nodes are strongly connected to each other leads to an underestimate of the modularity for strongly connected modular graphs, and an equally underestimated antimodularity for multipartite graphs, as compared to the measure *Q*_*H*_, when the number of modules is small. The functional measure *Q*_*H*_, in turn, cannot be used to *detect *the number of modules or communities, for precisely this reason: because no connection bias is assumed, there are no topological means to identify clusters. If the number of modules is given, on the other hand, *Q*_*H *_can be used to guide a graph partitioning algorithm. Note that the measures become indistinguishable in the limit of an infinite number of modules.

**Figure 8:**
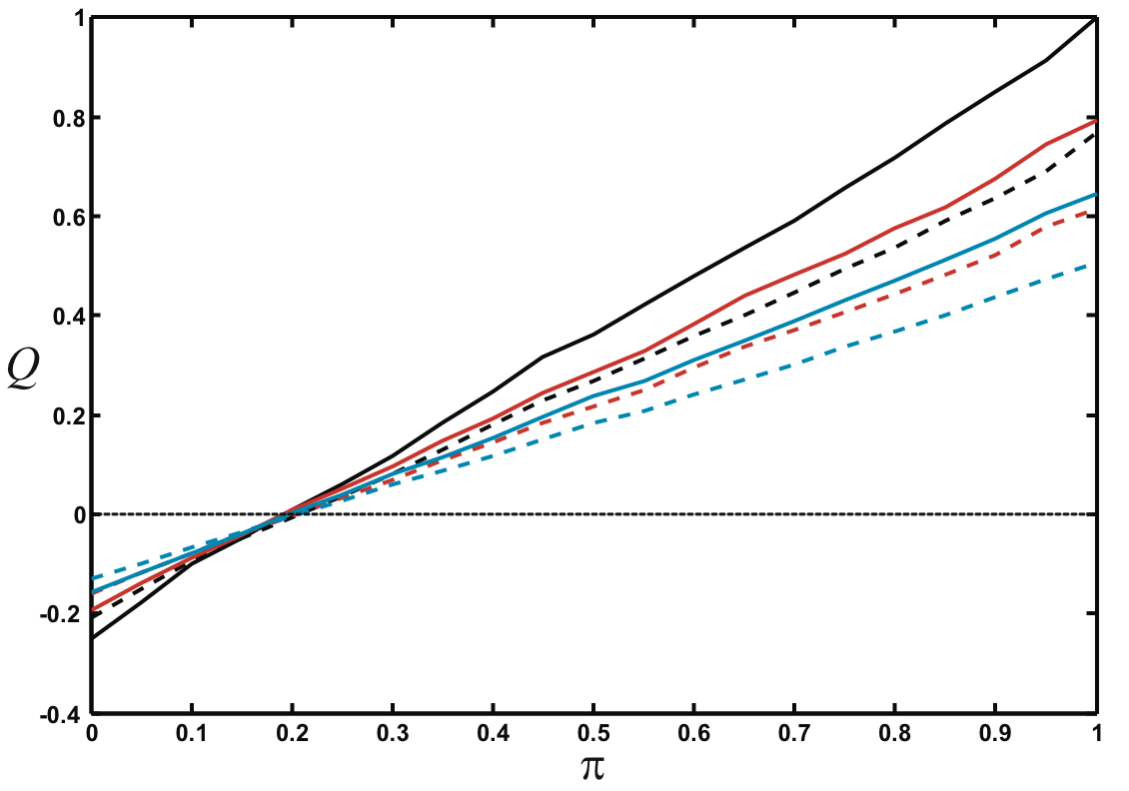
**Modularity depends on node-fusion probability**. Average modularity (from 50 independent networks with five modules, grown to 1000 nodes) for different node duplication probabilities *r *= 0.5 (blue), *r *= 0.75 (red), and *r *= 1.0 (black), for the modularity measures *Q*_*N *_(solid lines) and *Q*_*H *_(dashed lines). The networks were grown with the stochastic parameters set to *P*_*N *_= 0.5, *P*_*E *_= 1, *p *= *q *= 1.0, *P*_*D *_= 0.2.

We can also investigate the impact node *fusion *has on modularity (Figure 8), for the modularity measures *Q*_*N *_and *Q*_*H*_, by studying how modularity depends on module strength in networks grown with different node fusion probabilities. Irrespective of the measure, modularity is highest if nodes are not fused (*r *= 1) and decreases as the node fusion probability increases (*r *< 1) because node fusion is blind to the module assignment, while node duplication creates another node with the same color and the same edges as the original node. The larger the probability for adding an edge within modules is (larger *π*), the more modular the networks are, as expected. Because *Q*_*H *_does not penalize modules if they do not have an excess of edges between them, *Q*_*H *_is mostly larger than *Q*_*N*_. For small *π*, more connections exist between nodes of *different modules *than within them, so that both modularity measures become *negative*.

The impact of node *duplication *on modularity is more complicated. On the one hand, because node duplication brings with it the duplication of the edges that the duplicated node is attached to, whether or not node duplication leads to an increase in modularity depends on whether the network sports more inter-module or more intra-module edges. On the other hand, node duplication can skew the fraction of nodes that belong to any particular module by amplifying stochastic events that occur early-on in network growth. While node colors are chosen either randomly or according to a node probability vector when a node is created (see **Model**), the color of a node (that is, its module membership) is inherited under duplication. As a consequence, module *sizes *fluctuate considerably across different realizations of the network, and the modularity can become significantly different from that predicted by the *e*-matrix generating the network. A detailed analysis of duplication on modularity is beyond the scope of this manuscript, and will be presented elsewhere.

### Global properties

A number of interesting global topological properties have been observed in networks, both in the case of biological or engineering networks that are built via growth processes, and in Erdös-Rényi-type networks that form via random edge addition. Foremost in the first category is the "small-world" effect: the observation that many biological and technological networks have a short mean path between nodes (as compared to an equivalent randomized network), while being highly clustered (again with respect to a randomized equivalent network [52], see also the review [53]). Humphries and Gurney [53] introduced a quantitative measure to study the "small-world-ness" of a network, which is particularly useful because networks that have a high edge-density can automatically appear to be in the small-world class, but trivially so. Following Humphries and Gurney, we define the ratio of the "mean shortest path between nodes" in a network to the mean shortest path in the randomized version of the network (an Erdös-Rényi network with the same number of nodes and edges):(13)

and the ratio of the graph clustering coefficient  with respect to that of the randomized version (14)

The symbol Δ in the superscript of the clustering coefficients serves to remind us that this coefficient is obtained by counting the number of "triangles" of nodes normalized to the number of pairs [54], which can be different from the clustering coefficient obtained by averaging the number of edges of the adjacent nodes [52].

As small-world networks are identified by having a large *γ*_*g *_and a small *λ*_*g*_, the ratio of these quantities can be used to measure small-world-ness:(15)

**Figure 9:**
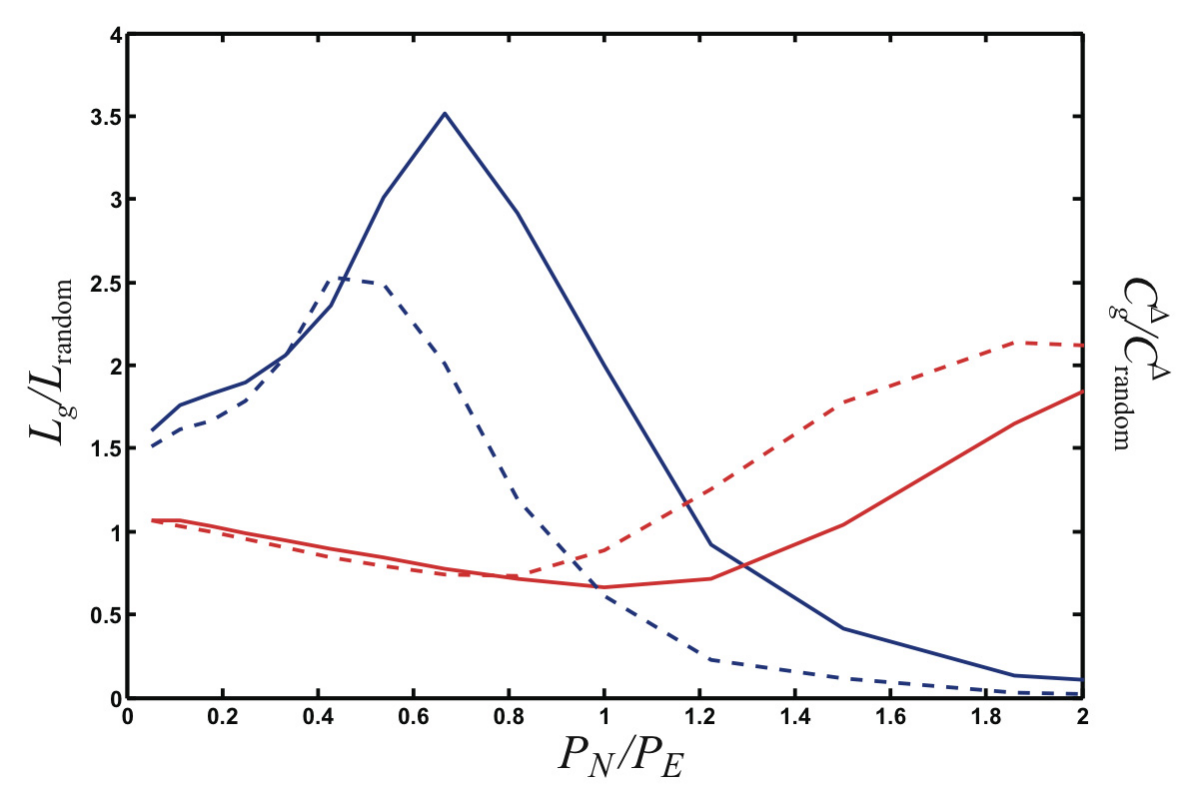
**Normalized mean shortest path and correlation coefficient**. The normalized mean shortest path between nodes, *L*_*g*_/*L*_random _(red lines) and the normalized correlation coefficient  (blue lines) as a function of the ratio *P*_*N*_/*P*_*E*_, for a node duplication probability *p *= 1 and two different edge addition probabilities *q *= 0.75 (dashed) and *q *= 1 (solid). Average over 1,000 networks grown to 200 nodes, with *P*_*D *_= 0.

We show the behavior of *λ*_*g *_and  in Fig. 9 as a function of the ratio of node- to edge-event probability *P*_*N*_/*P*_*E *_for networks grown to a size of 200 nodes. As more and more nodes are added per edge-addition event (increasing ratio *P*_*N*_/*P*_*E *_towards 1), the normalized mean shortest path first drops, and indeed, as long as *P*_*N*_/*P*_*E *_< 1.5 (for *q *= 1), the shortest paths in these networks are *shorter *than those in randomized networks. But once passed this threshold, the addition of more nodes without a commensurate increase in edges leads to longer and longer shortest paths (see Fig. 9, solid red line). The normalized correlation coefficient increases rapidly with an increasing ratio *P*_*N*_/*P*_*E *_up until *P*_*N *_≈ 0.65*P*_*E*_, after which the ratio drops very fast.

We also tested how decreasing the conditional edge-addition probability *q *affects *λ*_*g *_and . We expect that a decrease in *q *will move the mean shortest path and the correlation coefficient towards their randomized graph equivalents, because a *q *< 1 implies that sometimes edges are removed (for *q *= 1/2 edges are added as often as they are removed during an edge event), and the edge removal/edge addition process is tantamount to a *randomization *of the graph. We do indeed observe that  → *C*_random _as *q *decreases (we show the case of *q *= 0.75 in Fig. 9), but *L*_*g*_/*L*_random _actually *increases *for decreasing *q *as long as *P*_*N*_/*P*_*E *_≲ 2.

**Figure 10:**
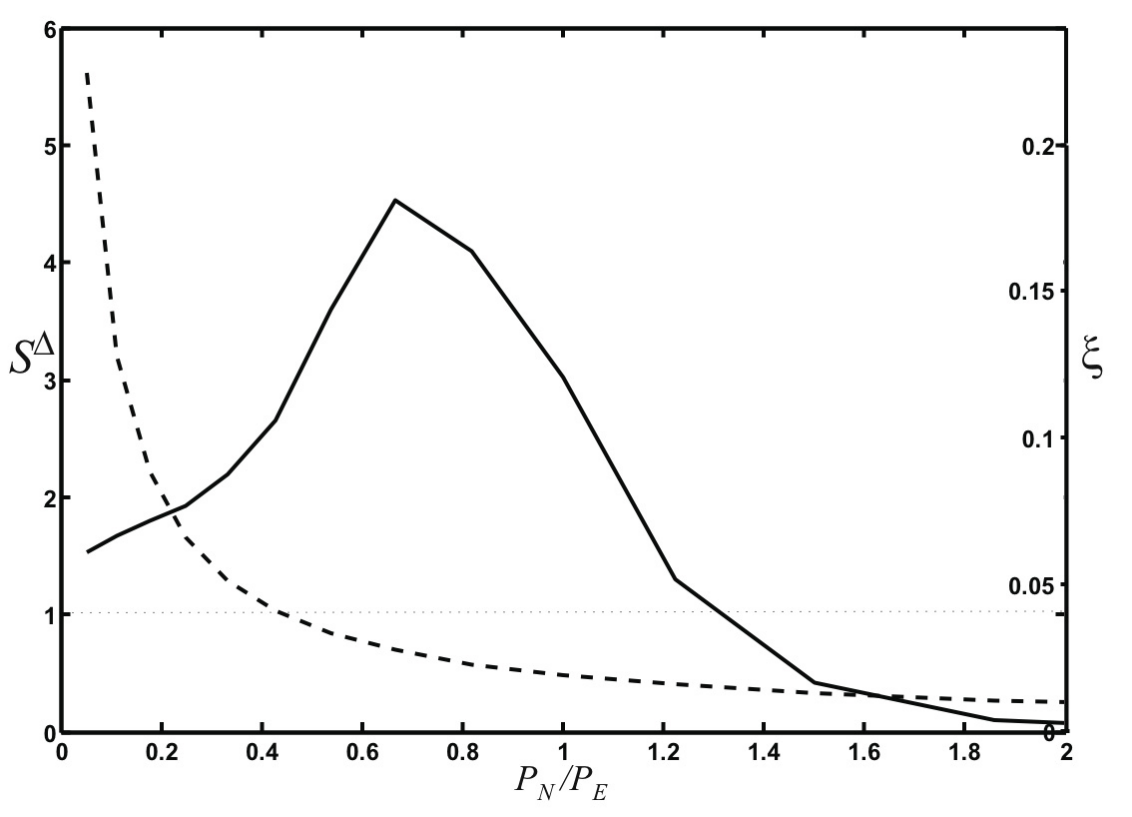
**Small-world-ness *S*^Δ ^as a function of *P*_*N*_/*P*_*E*_**. The quantitative measure of small-worldness *S*^Δ ^(solid line) and the edge density *ξ *[Eq. 16] (dashed line) as a function of the node to edge addition probability ratio. Networks are non-trivally "small-world" if *S*^Δ ^> 1 while the edge density is low (e.g., *ξ *< 0.1).

In order to determine what graph-growth parameters give rise to small-world networks, we plot the ratio *S*^Δ ^defined in Eq. (15) (which is just the ratio of the two curves depicted in Fig. 9) as a function of the ratio *P*_*N*_/*P*_*E *_(Fig. 10). In this figure, we also plot the edge density (or "sparseness") of the network (here *m *is again the number of edges, and *n *the number of nodes in the network)(16)

because it is known that networks with a high density of edges can be trivially of the small-world kind [53]. We see from Figs. 9 and 10 that networks grown with a ratio *P*_*N*_/*P*_*E *_< 1.3 have a small-world character, and furthermore that this character is maintained even for small ratios *P*_*N*_/*P*_*E *_down to about *P*_*N*_/*P*_*E *_≈ 0.2, where the edge density is *ξ *~ 0.1. This behavior is similar to that observed in the Watts-Strogatz model [52], which becomes "trivially small-world" in the limit of increasing randomness [53].

Figure 10 suggests that networks with small-world character are an automatic by-product of a stochastic growth process where the edge-event probability is of the order of the node-event probability or larger (here, *P*_*E*_/*P*_*N *_≳ 0.75), while the small-world character becomes trivial if *P*_*E *_is many times *P*_*N*_. This is a plausible scenario for a number of biological networks, where it is much more likely to create a new edge (for example, an interaction between two proteins via a gain-of-function mutation, or a regulatory interaction) than it is to create a new node (the evolution of a new protein *de novo *or via lateral gene transfer). We can also see that this regime is easily achieved in social networks, as long as the creation of an interaction between nodes is more common than the addition of a new member to the social network.

### Critical behavior

**Figure 11:**
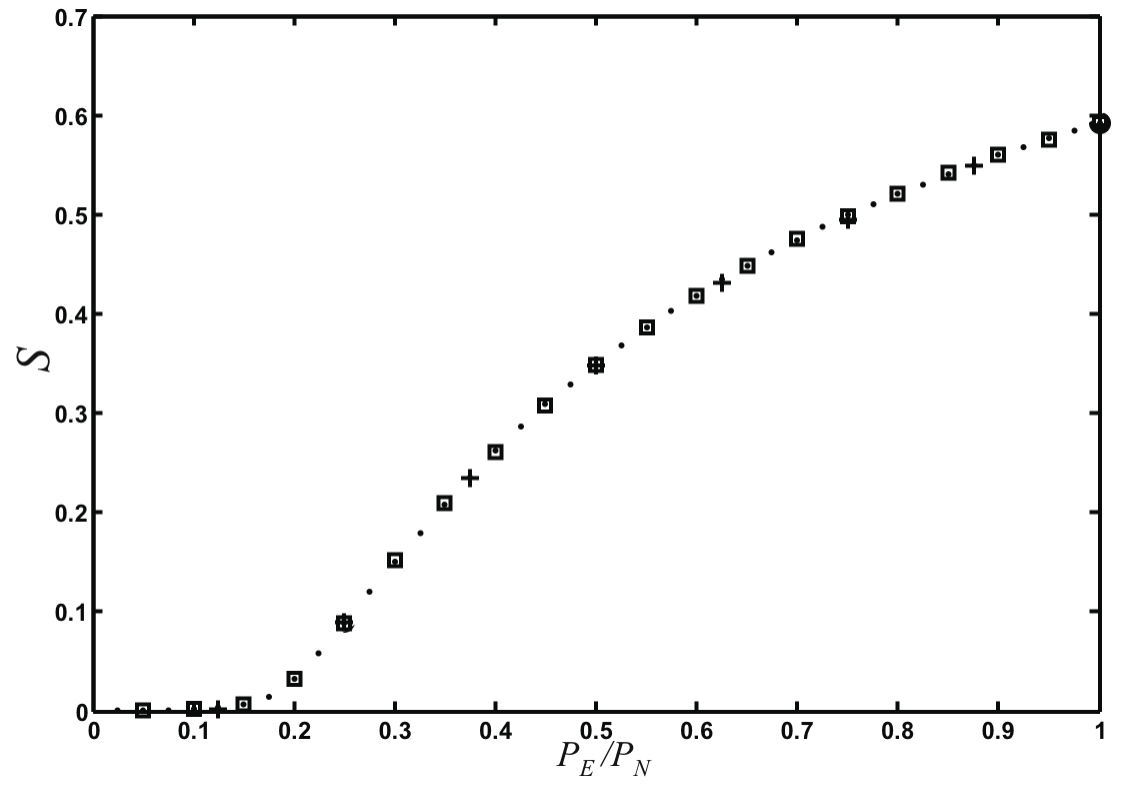
**Percolation phase transition in randomly grown networks**. Relative size of the giant component *S *as a function of *P*_*E*_/*P*_*N*_, for networks grown with various combinations of *P*_*N *_and *P*_*E*_: Crosses: *P*_*N *_= 0.1, circles: *P*_*N *_= 0.2, squares: *P*_*N *_= 0.5, dots: *P*_*N *_= 1.0 (with *P*_*D *_= 0 and *p *= *q *= *r *= 1). Networks grown to 10,000 edges, average over 100 networks.

Besides degree distribution, modularity, and small-world-ness, a number of other global properties of networks have been studied in the literature that we can study with ease using our network growth model. It is known since the pioneering work of Erdös, Renyi, and Bollobas that static random graphs undergo a phase transition where a "giant component" (a large connected component that scales with the size of the system) emerges at a critical probability of connecting edges (see, e.g., [55]). This phase transition is of the same kind as in percolation models, and is often referred to as the percolation transition in random graphs. Callaway et al. [43], Dorogovtsev et al. [56], as well as Bollobas et al. [57] have pointed out that random networks that are grown also undergo a percolation transition, but that this transition has a very different character: the critical point is infinitely differentiable (in fact, all the derivatives vanish at the critical point [43,56,57]). We can study this transition in our model as a function of parameters not previously investigated, namely *P*_*N*_, *p*, *q*, as well *P*_*D *_and *r*. We observe that the percolation phase transition only depends on the ratio *P*_*E*_/*P*_*N *_(see Fig. 11), that is, the size of the giant component *S *only depends on the *relative *rate at which nodes and edges are added. Even varying *q *(allowing for edge removals) does not change this transition, as long as we plot the giant components versus *qP*_*E*_/*P*_*N *_instead (results not shown). Note that this combination of parameters is related to the asymptotic mean degree ⟨*k*⟩ (see Eq. (5) in **Models and Methods**).

**Figure 12:**
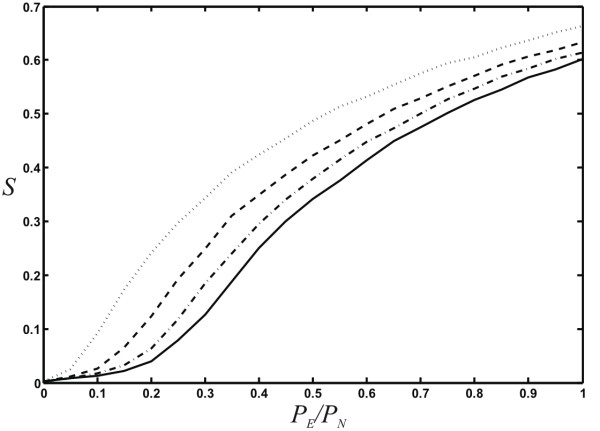
**Percolation phase transition with node fusion**. The relative size of the largest connected component *S *as a function of the relative edge to node event probability (with *p *= 1, *q *= 1, and *r *= 0), for different node fusion probabilities (as *r *sets the node duplication probability, the fusion probability (1-*r*)*P*_*D *_is simply given by *P*_*D*_). Solid line: *P*_*D *_= 0, dash-dotted: *P*_*D *_= 0.1, dashed: *P*_*D *_= 0.25, and dotted: *P*_*D *_= 0.5. Average of 100 replicates of networks grown to size *n *= 100.

Similarly, allowing for node duplication does not change the transition, as duplications may change the absolute size of the giant component, but do not affect its emergence. Node *fusion*, on the other hand, does affect the emergence of the giant component because fusions can lead to the merger of two separate clusters. We show in Fig. 12 the relative size of the largest connected component of networks grown with different node fusion probabilities (*P*_*D *_= 0.0, 0.1, 0.25, 0.5, *r *= 0), where the value *P*_*D *_= 0 serves as the control (no node fusion). As expected, the onset of the transition is earlier when nodes can fuse, because node fusion does not change the giant component if the nodes are in the same cluster (except for diminishing its size by one), whereas whole clusters are fused if the nodes that are fused belong to different clusters. The nature of the phase transition (infinitely differentiable critical point) is unchanged. Of course, as nodes are fused, the network grows more slowly, but the shape of the curve in Fig. 12 cannot be recovered simply by scaling *P*_*N *_and *P*_*E *_taking into account the modified number of nodes and edges for each fusion event (data not shown).

### Biological relevance

Given the variability of the networks that can be generated with this model, we may ask whether it is universal in the sense that the edge distribution of *any *biological network can be characterized by the set of parameters (five independent constants plus the *e*-matrix). We tested whether networks can be grown to have an edge distribution that is similar to well-known biological reference examples, and whether the set of parameters giving rise to these networks allows us, by analogy, to generate hypotheses about the process that generates them. Specifically, we grew networks to resemble the edge distribution of the neuronal network of the nematode *C. elegans *[58], as well as a network similar to the protein-protein interaction network of yeast [59]. The best current data set for the *C. elegans *"brain" includes 280 of the 302 neurons and their connections [58]. We binned the edge distribution from this data set and searched the parameter space of the model (five parameters, no modules, undirected edges) for sets that grow networks of 280 nodes with an edge distribution that minimizes the root mean square difference of the corresponding binned edge distribution. Note that because a graph's properties are unchanged if the relative ratio of the three event probabilities is maintained (as long as neither of them becomes too small), we kept the largest of the parameters (here *P*_*E*_) fixed. We verified that a search with six independent parameters gives rise to the same set if rescaled to *P*_*E *_= 1.

**Figure 13:**
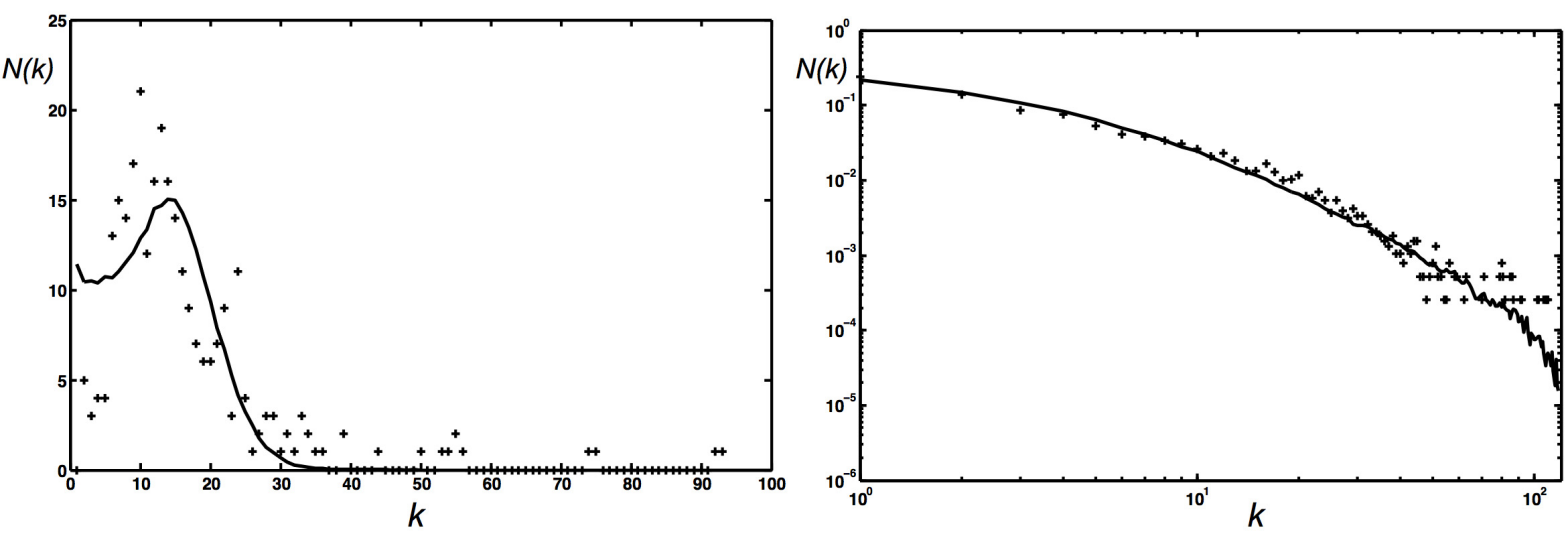
**Edge distributions of actual and simulated biological networks**. (A) Edge distribution of a network optimized to reproduce the edge distribution of the *C. elegans *neural network (solid line), compared to the observed distribution ("+"). (B) Edge distribution of a network optimized to reproduce the edge distribution of the yeast protein-protein interaction network (solid line), compared to the degree distribution reported in Ref. [59] ("+"). The eight proteins with the highest degrees have been removed for the purpose of this comparison because they do not follow the distribution of the remaining proteins.

Within the space of network parameters, the *C. elegans *network appears to be fairly rare, so that a straightforward Monte Carlo search often arrives at inferior fits. Our best solution is a network with *P*_*N *_= 0.008, *p *= 0.71, *P*_*E *_= 1.0, *q *= 0.06, *P*_*D *_= 0.028, *r *= 1.0 (*P*_*E *_was fixed at 1.0 in this search). We show the degree distribution generated with this set of parameters (averaged over 1,000 realizations of the network) in Fig. 13A (solid line). A statistical test comparing these distributions cannot reject the hypothesis that they were generated from the same underlying probability distribution (Wilxocon rank sum test, P = 0.744). This set of probabilities suggests that the *C. elegans *network reflects a growth process with a very small node addition probability, commensurate with our earlier observation that networks with an Erdös-Rényi-like degree distributions are obtained using a small *P*_*N*_. Such a small node addition probability is also consistent with the constraints imposed on *C. elegans *evolution by its invariant cell-lineage. The worm develops via *stereotyped cleavages *so that the patterns of cell division, differentiation, and death are the same from one individual to another: in the developing worm each cell has a predictable future, and each cell a well-defined set of neighbours [60]. As a consequence, developmental changes giving rise to new nodes must be heavily constrained, as they would upset the delicate balance. For the same reason, node duplications are also virtually absent in the simulated network. The small edge addition probability *q *= 0.06 implies that edges are only added in 6% of the edge events, and the network is consequently quite sparse. As the algorithm does not remove an edge if there is none between the randomly selected nodes, even such a small edge addition probability (in fact, it corresponds to a 0.94 edge *removal *probability) always gives rise to an equilibrium edge count (calculated in **Models and Methods **in the absence of duplication).

The simulation of the yeast protein-protein interaction network (interaction data from the highly curated set of Ref. [59]) leads us to vastly different conclusions concerning the nature of the growth process. Because this network is much less rare, a Monte Carlo search converges fairly rapidly, and yields a similar set of parameters in all trials. For yeast, we started with 4 different initial conditions, conducted 5 trials each, and grew 20 replicas for each parameter set to obtain an average distribution, which we score by comparing the root mean square difference of the binned distribution to the binned yeast distribution. Because of the sparseness of the data at high degrees, we performed a "threshold-binning" with variable bin size, as described in [61]. We stop growth at 3,306 nodes (the size of the Reguly et al. network) and obtain a network that is remarkably similar to the yeast protein-protein interaction network, with *P*_*N *_= 0.7 ± 0.04, *p *= 1.0 ± 0.05, *P*_*E *_= 1.0 ± 0, *q *= 0.91 ± 0.035, *P*_*D *_= 0.75 ± 0.035, *r *= 1.0 ± 0.03. A statistical test comparing the yeast degree distribution and that of our simulation for nodes with less than 120 edges (see Figure 13B) cannot reject the hypothesis that both distributions were obtained from the same underlying process (Wilcoxon rank sum test, P = 0.0837). The eight proteins in the yeast network with more than 120 nodes appear to be outliers that do not follow the same law as the remaining 3,298 proteins in the network.

Both the node addition probability *P*_*N *_and the node duplication probability *P*_*D *_creating our surrogate yeast network are remarkably high. That the node duplication probability is this high for the generation of a protein-protein interaction data set is not surprising in the light of evidence that much of genomic evolution proceeds via gene duplication and subsequent diversification [62,63]. However, the analysis also suggests that the yeast network edge distribution is only very approximately scale-free.

We conclude that particular degree distributions are (at least for the cases we examined) obtained with unique parameters sets, thus allowing us to entertain hypotheses about the processes that generated the networks we are simulating. Of course, such speculations rest on the assumption that other processes (such as for example, whole genome duplication, or horizontal transfer of sets of genes) do not play a role in shaping the network's edge distribution. Because we cannot rule out such processes in many of the standard networks, such a caveat always has to be issued.

## Conclusion

We have presented an algorithm that, using only a few parameters, can generate networks of seemingly arbitrary degree distribution, modularity, and structure. Using this model, we were able to study how fundamental properties such as edge addition or removal, as well as node addition, removal, duplication, or fusion, affect a network's degree distribution, modularity, and structure. We found that we could grow networks with degree distributions anywhere between binomial, exponential, and scale-free, within a single framework or process. By introducing an assortativity matrix for the generation of nodes with different functional tags, we could furthermore grow networks with different degrees of modularity, by specifying the probability that a node of one "color" will attach to a node with a different color. Once modules or functional groups have been identified in any real network, control networks can be grown that mimic the connection pattern or modularity of that network. One obvious example is again the *C. elegans *neuronal network. Its nodes can be divided into three classes: sensor neurons, motor neurons, and interneurons [58]. The *e*-matrix for this network can be reconstructed from the frequencies of inter-color edges, and be used to grow networks that not only have the same degree distribution as the original network, but mimic the connection pattern between functional types as well.

We also introduced a modified modularity measure *Q*_*H *_for networks that is based entirely on the functional characterization of a network's nodes, rather than the connection pattern. This measure is neither better or worse than any of the existing modularity measures (such as Newman's *Q*_*N *_or the assortativity *r*), but rather highlights a different aspect of modularity. For example, while Newman's modularity *Q*_*N *_defines modules as those groups of nodes that are connected to each other more often than would be expected from the connection probability in a random graph of the Erdös-Rényi-type, the measure *Q*_*H *_does not assume such a bias. In fact, because most biological, social, and technological networks are not of the Erdös-Rényi-type, it is often erroneous to compare the connection probability of modules to what would be expected in a random graph. This is particularly true for networks with scale-free edge distribution, which sport a number of hubs with many edges that do not necessarily connect to other nodes within the same module. The measure *Q*_*N *_will attempt to join such hubs in one and the same module, even if a measure based on betweenness centrality will separate them (see Appendix). *Q*_*H*_, in contrast, does not allow you to detect modules, but rather quantifies the modularity of a graph based entirely on a previous group identification. Using such a measure, we can show that graphs are often less than modular, and can even become anti-modular. An extreme case of anti-modularity is given by bi-partite, and by extension, *k*-partite graphs. In fact, precisely modular and *k*-partite graphs appear as "dual opposites" in this framework, obtained with an *e*-matrix with only ones on the diagonal and zeros elsewhere (divided by the number of modules), or else zeros on the diagonal for the *k*-partite graphs.

The networks created using the present model recapitulate a large swath of existing literature concerning networks, of which we presented a selection here. For example, we were able to study how to generate networks with given global properties, such as small mean distance between nodes, or high clustering coefficient, and by extension examine the nature of small-world graphs. We were also able to study the percolation phase transition in networks, but unlike in the standard literature where the probability that edges are connected given a fixed set of nodes (or, as in Ref. [43], the node addition probability is fixed at *P*_*N *_= 1), we were able to study the size of the "giant component" as a function of the ratio of the edge to node addition probability, and found the same phase transition. In addition, we could study the effect of node duplication and/or fusion on the nature and location of the transition.

Finally, we used the model to reverse engineer the growth parameters that might have led to the observed degree distributions of the *C. elegans *neuronal connection graph and the yeast protein-protein interaction network, while keeping in mind the assumptions behind that extrapolation. A Monte Carlo search process through the five-dimensional parameter space (not using modules) converged to suggest a unique set of parameters for each of those networks that led to biological conclusions compatible with our current knowledge about the forces that shaped these networks. We expect this model to be most useful in the generation of null models in the analysis of biological, technological, and social networks. The process easily generates networks with the same size and degree distribution as any study network, but unlike the method presented in Ref. [64], the present process relies explicitly on network growth and can accommodate arbitrary node "colorizations" (functional categories). Another standard control, the edge randomization of any network, is easily implemented by setting *P*_*N *_= 0, *P*_*E *_= 1, *q *= 0.5 (with *P*_*D *_= 0). This setting will remove and place edges randomly while keeping the total number of edges and nodes the same, resulting in a randomized graph after a sufficient number of updates.

We have shown that a broad set of standard results in network analysis, concerning the edge distribution, modularity, global network structure, and critical behavior, can be reproduced in networks grown via a random process, with only a few tunable parameters. These networks, however, were grown in the absence of any functional constraint, and their properties are therefore a consequence of the stochastic nature of the growth process only. We can conclude that while such properties may be useful for real-world networks, they are not necessary consequences of the network's functionality, but could simply be a consequence of how they emerged.

## Competing interests

The authors declare that they have no competing interests.

## Authors' contributions

AH and CA conducted the research and wrote the manuscript.

## Appendix

### Structural and functional modularity

Newman's modularity measure Eq. (8) can be shown to be related to his assortativity measure (9) by noting that the mixing matrix *e *is related to the adjacency matrix *A *via a transform involving a matrix *F *that relates nodes to modules:(17)

Introduce(18)

Because , we find that(19)

where the notation ||...|| indicates taking the sum of all the matrix elements. The mixing matrix *e *is then just(20)

Noting that Tr*FF*^*T *^= *S*, the modularity matrix defined above (8), we find that(21)

The same construction also allows us to write *Q*_*H *_[defined in Eq. (11)] in terms of Tr *e *[Eq. (12)] by noting that(22)

where **1 **is a matrix where each entry is 1.

**Figure 14:**
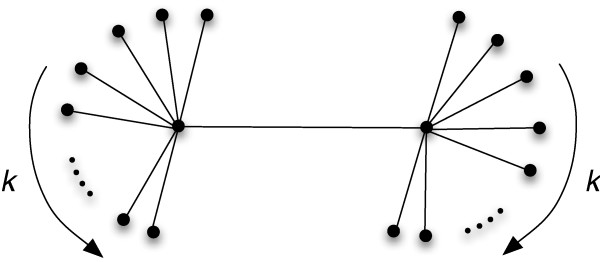
**A graph with two hubs and *k *edges per hub**.

We can test the limits of the modularity measures (8) and (11) by calculating the modularity of an extreme graph as depicted in Fig. 14, which is a graph of two hubs of degree *k *connected by a single edge, and assuming that all of the nodes of one hub belong to the same module. In the limit of large *k*, such a graph should be classified as highly modular. However, Newman's measure applied to this graph gives(23)

In comparison, the functional modularity measure *Q*_*H*_, making the same assumptions about the modules, tends to 1 in the limit of high-degree hubs:(24)

### Functional modularity and assortativity

Newman's assortativity *r *[Eq. (9)] and the functional modularity measure *Q*_*H *_[Eq. (11)] are identical for a particular assortativity model, which we call the "equal opportunity" model. It is defined by the probability *π *for a color to connect to a node of the same color, but to connect to any other color with equal probability:(25)

The factor 1/*N*_*c*_, where *N*_*c *_is the number of colors, serves to normalize *e *such that ||*e*|| = 1. It is easy to see that the fraction of nodes connected to type-*k *nodes: *a*_*k *_= ∑_ℓ _*e*_*k*ℓ _= 1/*N*_*c*_, so that (with Tr *e *= *π*)(26)

which agrees with *Q*_*H *_defined in Eq. (12).

## Reviewers' Comments

### Reviewer 1: Erik van Nimwegen, University of Basel

This paper introduces an interesting parameterized class of stochastic network growth models that clearly can produce networks with a range of different topological properties, i.e., degree distributions, modularity, mean path lengths, clustering coefficients, as the parameters of the stochastic growth process are varied. It is quite conceivable that this parameterized family of growth models might actually be used to capture the broad topological properties observed in many 'real world' networks. This is very nice. However, what is a bit disappointing is that there are almost no analytical results on precisely what kind of properties the networks will have as one varies the parameters. All that is presented is a number of 'anecdotal' examples of what one obtains with particular parameter settings. Moreover, the results are complex enough that one cannot easily generalize from the examples presented. That leaves us in the end in a state where we have a family of growth models that MIGHT produce networks with desired properties, but it is unclear which kind of properties can be produced and precisely how to set the parameters to get them. That is, if I were to say: I have this real world network and it has this degree distribution, this distribution of distances between nodes, this distribution of clustering coefficients, and this modularity (according to whatever measure), then I don't think the authors could tell me whether there model could produce graphs with the same properties, and how to set parameters to get such graphs. In fact, the last section of the manuscript shows that, even to reproduce only the degree distributions of two biological networks, an expensive Monte-Carlo search is needed, and in the end the results clearly suggest that the growth model in fact cannot reproduce the observed degree distribution within statistical noise. This to me suggests that the practical use of this family of growth models is extremely limited until more general theoretical results about their behavior can be obtained.

**Authors' response**: *First, we would like to thank you for the time you have spent reading our article, and assembling a long and detailed set of comments and questions. Many of your comments have led to important improvements in the presentation of the material. One of your main criticisms in the passage above, and repeated elsewhere in your comments, is a disappointment over the lack of a mathematical analysis of the graph growth model that we studied computationally. We have a good amount of sympathy for this position: it would be great to have a mathematical model that does all the things that you mention, and more. The problem with this is, also pointed out by you, that there are an infinite number of possible graphs, and there is simply no generative theory that could account for them all. Now, your suggestions for a mathematical theory sound less ambitious than that: you ask whether it would be possible to produce analytical results that will predict the form of the edge distribution, for example, given the input parameters, in the limit of infinite network size. This is at face value a reasonable proposition: after all, there is literature that predicts just that for a class of models that is a subset of the model described here. For example, it is possible to calculate the asymptotic properties of graphs produced by a duplication model *[41]. *But we wonder whether you are fully aware of how ambitious this proposition is. After all, deriving just the asymptotic degree distribution for a pure duplication model as in *[41]*(which has a single parameter) is a ten-page publication. We have six main parameters (five of them independent, we will come to that), and possibly an infinite number of other parameters that allow us to specify certain other aspects of the graph (such as the modularity) and even the adjacency matrix if we so desired. It is not that having a mathematical analysis of the sort that you wish you had seen would not be worthwhile having, it is just not the direction we chose to go because this is a tremendous undertaking that would take years of additional work. We have instead taken a much more practical approach. We understand that, for real systems, we will never know for sure what growth parameters have given rise to that network: even if we reproduced a particular biological network perfectly in all those properties that we can measure, we still could not state with certainty that these actually were the parameters that gave rise to the biological or technological graph. As we are forced to give up this goal, we determined that it was going to be much more useful to have a model that can generate networks that are approximately like those that we observe, even if there is no proof about any of the observed properties. At the same time, many of the qualities you look for in a mathematical theory are completely irrelevant if you are analyzing real-world graphs. For real-world applications, you are never interested in the infinite graph size limit, you will never see *"*pure" power laws (these hold for infinite systems only), you will never see a *"*true" geometric phase transition (again because networks aren't infinite), and you will also never see a *"*true" Poissonian degree distribution either. The practical issues you face when analyzing real networks are for example: *"*What is a good control graph for the hypothesis I'm testing?" and *"*Why do some networks have an approximately scale-free degree distribution, and others not?" The purpose of our work is to create a model that allows the user to test hypotheses about network generation. We have introduced language in the introduction of the manuscript to make this purpose more obvious*.

To become a bit more specific, apart from a general theory on how the topological properties depend on parameter settings, what I thought was really missing is a treatment of how things depend on the final size of the network and on the initial conditions.

**Authors' response**: *The dependence of networks on the initial conditions is indeed an interesting question, but in the light of the response above, also not within the scope of our ambition. The *"*envelope" created by the set of all possible starting conditions for network growth is tremendous. In almost all of our simulations, we have used a single node as initial condition. In simulations without modules and without duplication, it is clear that we do not need to investigate the case of several nodes and edges as starting conditions, as they represent possible later stages of the graph in the growth procedure. We note that this is not true if we grow modular graphs (that is, when nodes have colors denoting different functional groups). The probability that nodes can be duplicated leads to a strong dependence of the final modularity of the graph on initial conditions that is difficult to predict mathematically. As a consequence, we have deferred this analysis to a subsequent publication*.

Then there are also subtler issues such as whether the statistics of the networks grown are self-averaging, i.e., do different instantiations of the same stochastic growth process always lead to networks with the same features (degree distribution, modularity, path lengths, et cetera) or might one have different 'attractors' where sometimes you will get networks of one kind and sometimes of another kind? As far as I can see none of these issues are discussed at all in the manuscript. Apparently the authors are assuming that the observed features (degree distributions etc.) do not depend on either the initial condition or on the final size of the network. However, it is not obvious to me at all that these statistics are independent of how long the network has been grown (in fact, I doubt it is for all possible combinations of parameters). Thus, either the authors should provide an analysis of how network features depend on final size and initial conditional, or they should present some general theory showing why (maybe in the limit of large networks) the features become independent of size and initial condition.

**Authors' response**: *This is a valid criticism: we did not discuss the self-averaging properties of the graphs we generate. We do not have any mathematical results to offer, nor did we perform an exhaustive analysis of the dependence of the graph properties on the size of the grown network, that is, the length of the growth process. The reason we did not pursue this is simple: Some properties depend on the size of the network, some others do not. Edge distributions become stationary fairly quickly as you surmise. Other properties, such as the critical properties of graphs, become more pronounced, as is also obvious without doing a detailed analysis. We did, however, check that the relative size of the *"*giant component" that shows the percolation transition in randomly grown graphs *(Figure 11) *approaches the limit found in Ref*. [43]*for graphs ten times larger than the one shown in that Figure. In fact, the curves are indistinguishable to the naked eye already at the size we show. In so far that we have observed that properties that we discuss to become independent of the length of the growth process, we now explicitly mention this in the text. Of course, we understand that this falls short of displaying an extensive analysis or providing a general theory, but we surmise that it would satisfy most readers without producing an absolutely unwieldy manuscript*.

In the remainder of the review I will make more detailed comments on specific statements in the manuscript. First about some statements in the abstract:

"...but is created artificially and therefore carries none of the function...". I do not agree with this. It is well conceivable that part of the function may actually reside in these features so that any surrogate network that carries these features would automatically carry some of the function.

**Authors' response**: *We believe this is a misunderstanding. We do not imply with this statement that the networks we create would be non-functional if somehow instantiated in a biological setting. We simply mean to say that in our simulation, the growth process does not underlie any selective pressures related to a functional constraint. Therefore, we can test a hypothesis that a particular feature of a network necessarily requires a particular functional constraint to be present during growth. We have changed that sentence to clarify what we meant to say*.

"we present an algorithmic model for growing networks with arbitrary degree distributions... using a small set of parameters." This claim is made over and over in the manuscript and it really started getting on my nerves. It namely suggests that the authors fail to grasp the basic mathematical fact that one cannot possibly reproduce ARBITRARY degree distributions (which form an infinite dimensional space) using a family of processes that has only a small number of parameters (a 5-dimensional space). While the model presented by the authors may reproduce many of the degree distributions of interests (e.g. exponentials and power-laws), it is certainly false that it produces 'arbitrary' degree distributions. In fact, all results in the paper suggests that the space of possible degree distributions that it produces is in fact fairly limited. It can produce exponentials and power-laws (not even that precisely it seems), but over what range of exponents? Can it generate stretched exponentials? More general Zipf's distributions? Gamma-distributed degree distributions? Lognormal ones?

**Authors' response**: *As you realize later on in your report, we did not *"*fail to grasp the mathematical fact that one cannot reproduce arbitrary degree distributions (...) using a family of processes that has only a small number of parameters", because we can indeed prescribe any degree distribution using the assortativity matrix. We understand this criticism to mean that we do not issue enough of a forceful caveat that our five independent parameters alone, without using the generative capacity of the assortativity matrix, cannot reproduce arbitrary degree distributions. This is a point well taken, and we have included language to this effect in the abstract and the introduction*.

"We find that many of the celebrated network properties may be a consequence of the way in which they grew, rather than a necessary consequence of how they work or function." It would be really really nice if this were established in this paper but I frankly do not think that this manuscript actually does anything of the sort. To do this one really would have to show that 'function' is in a sense orthogonal to these properties, i.e. that the required function does not require these properties and that these properties have no functional implications. But 'function' is nowhere studied in the manuscript.

**Authors' response**: *I think we disagree here. Our statement is really very modest: We claim that we can show that a particular network property MAY be a consequence of how it grew. We are aware that our model can only falsify hypotheses about the necessity of a property (such as: *"*scale-free degree distributions can only arise if...."). In other words, we simply claim that other processes than functional constraints (such as growth) can be responsible for a form. Instead, you are reading our statement to falsify the claim that a particular function implies a graph property (form), which would require us showing that absence of form implies absence of function. But our statement is really clear about what we mean, so we see no need to modify it. We fully understand that just because a graph property arises without a functional constraint, this does not imply that it could not be useful. That would be silly*.

page 2: "often information external to the purely topological structure is used to determine modular relationships, such as co-regulation or evolutionary conservation." What I find confusing in this statement is that different researchers use different features to connect 'nodes' by edges, so some researchers may well draw a network based on co-expression of genes or on their evolutionary conservation. Therefore, whether this kind of information is 'external' or not to the topology is a matter of definition.

**Authors' response**: *Of course, any information that implies a relationship between nodes can be used to draw edges between nodes. But measures of modularity usually assume two kinds of connection matrices: the connection matrix of what node connects to another, defined for example by protein-protein interactions, and an assortativity matrix that determines which nodes belong to which functional cluster or group. When we talk about information external to the topological structure, we talk about information that enters the construction of the assortativity matrix, not the topology matrix*.

"in the limit of a large number of nodes, approximately Poisson." Formally another requirement is that the expected number of edges per node does not grow with the total number of nodes in the graph, i.e., the probability for an edge to exist scales as *r=n *with *n *the number of nodes in the graph.

**Authors' response**: *In this sentence, we discussed what other authors found when studying random networks in the limit of a large number of nodes, not what their requirement was to call a particular distribution a Poisson distribution*.

"While random networks can be found in social...." This is a detail but I find the use of the term 'random' confusing here. What the authors are presumably referring to is graphs with particular degree distributions, i.e. Poisson. Graphs with other degree distributions can also results from random processes. In fact, that's precisely the topic of this paper. There are other places where the authors use 'random graphs' where 'Erdosz-Renyi graphs' would be less ambiguous.

**Authors' response**: *We agree that we need to be careful not to use the same word when discussing graphs created by a random process, as opposed to random graphs defined by a Poissonian degree distribution. We have made sure that no such ambiguity remains in the text*.

"The emergence of this scale-free distribution can be understood in many different ways..." Maybe I can be forgiven for mentioning that, to the best of my knowledge, the first publication showing a scale-free distribution of degrees of a biological network (edges representing significant sequence similarity between genes in a genome) was by Martijn Huynen and myself [65]. A simple multiplicative noise model was also presented to explain the observed power-law distribution.

**Authors' response**: *Scale-free distributions are ubiquitous in nature because of the simplicity of the process that gives rise to them. In fact, any branching process where the rate of producing *"*similar" is much higher than the rate of producing *"*new" will lead to a power law. This has been known since 1924, when Yule applied such a model to understand the size distribution of taxon abundances *[66]. *A simple model that shows how violations of the scale-free distribution allow you to estimate the ratio between the parameters of the branching process is presented in *[61], *which also, incidentally, throws some light on the results in your MBE paper*.

"...are fundamentally different from those produced probabilistically". Again, this is a confusing use of the word 'probabilistic' because the growth models studied here are also definitely probabilistic (i.e., where and when an edge or node is added/removed is not deterministic).

**Authors' response**: *We agree that this language is imprecise. We have changed this sentence to say: *"*are fundamentally different from those where edges are placed between nodes probabilistically"*.

"...can produce networks with any degree distribution and any modularity..." As already mentioned, this is simply false. In fact, I think the authors would do readers a great service if they could show precisely what family of degree distributions can be obtained using their model. For example, on page 3 the authors claim that regular graphs and lattices can be generated using the model. At first I was confused by this but it is explained on page 5. However, what happens in this case is that one essentially encodes the entire graph in the assortativity matrix. In this limit one doesn't have a growth model at all anymore, one is really just specifying the entire adjacency matrix. Obviously, if I allow myself to specify an adjacency matrix I can get anything I want!

**Authors' response**: *As discussed earlier in this reply, it is true that the adjacency matrix is necessary in order to create truly arbitrary degree distributions, so technically our statement is not *"*simply false". However, we agree that some readers might be misled into thinking that arbitrary degree distributions could be obtained with five parameters only, and we therefore inserted language that makes this caveat clear. However, it is also not correct to say that if you specify an adjacency matrix using the assortativity matrix that no growth takes place. Rather, the network can still grow towards that degree distribution as nodes are added, or, if we start with a network fully-formed, the network can be shrunk by removing nodes and edges*.

The description of the model on page 3 is rather short and cryptic. It seems more natural to me to put the Models section simply before 'growing scale-free and other networks'.

**Authors' response**: *We just now discovered that this is indeed an option in the *"*Biology Direct" format (but not in other formats), and we have followed this suggestion. Thank you*.

I am also somewhat confused about the parametrization of the models. I initially assumed that *P*_*N *_+ *P*_*E *_+ *P*_*D *_= 1 but later I realized that these three probabilities are independent and this seems redundant to me. That is, if I start with a given setting of (*P*_*N*_, *P*_*E*_, *P*_*D*_) then I can divide all these probabilities by *X *(with *X *≥ 1) to get a parameter setting (*P*_*N*_/*X*,*P*_*E*_/*X*,*P*_*D*_/*X*) and it seems to me that this parameter setting would produce exactly the same networks.

**Authors' response**: *This is indeed correct. Because this X can be determined by any of the other probabilities, there are in fact only 5 independent parameters in this model (not counting those that go into the assortativity matrix). We were aware of this relation, but did not make it clear everywhere. We now added text that makes this relationship clear everywhere*.

This is in principle a small point but I noticed that in Figure 9 the authors show *S *as a function of *P*_*E*_/*P*_*N *_(*P*_*D *_= 0) for *P*_*N *_= 0.1, *P*_*N *_= 0.2, *P*_*N *_= 0.5, and *P*_*N *_= 1.0. Given that *P*_*E*_/*P*_*N *_is fixed, isn't it necessary that the different settings of *P*_*N *_just rescale time?

**Authors' response**: *The different settings of P*_*N *_*create the different ratios P*_*E*_/*P*_*N*_, *which dial the ratio between edges and nodes. We could just as well have left P*_*N *_*constant and varied P*_*E *_*(doing this creates the same exact S, as we have verified). So, no, P*_*E*_/*P*_*N *_*is not fixed*.

It seems to me a more logical parametrization is to think of 1 event happening at a time, and that this event can either be a node addition, edge addition, or duplication event, which occur with probabilities *P*_*N*_, *P*_*E*_, and (1 - *P*_*E *_- *P*_*N*_).

**Authors' response**: *We defined an update so that three events could take place in the same time step. Of course, the algorithm handles all events sequentially, so there is conceptually no difference. And because all the probabilities can be scaled by a common factor as you remarked*, "*time" is an arbitrary parameter anyway*.

What is extra confusing about this is that, toward the end of the paper, the authors do seem to acknowledge that there are only 5 independent parameters (whereas previously they claimed there are 6).

**Authors' response**: *We have now made the interdependence clear throughout the manuscript*.

page 3: "older nodes have a higher probability of obtaining edges, and also have a higher probability of already sporting more edges." This is confusing. Whenever a single edge is added an older node is equally likely to be chosen as a younger node. In fact, if the older node has more edges already (and typically of course they will) then there is actually a bigger chance that the edge addition will be UNSUCCESSFUL (because an edge is picked that already exists), so that effectively older nodes have a SMALLER probability of obtaining another edge. What is correct is that older nodes have had more opportunities to grow edges and will thus in general sport more edges than young nodes.

**Authors' response**: *Thank you for this clarification. We changed the wording according to this comment*.

"the scale-free distribution is unavoidable." This nowhere demonstrated. In fact none of the distributions shown in figure 1 are true power-laws. Also, as already mentioned, it is unclear what the limiting degree distribution is for large network size (and whether there is such a limit). It is also unclear if one can truly get power-law degree distributions for any parameter setting. Some theory would be required for this.

**Authors' response**: *First, true power laws do not exist for finite networks. It is, however, correct that we do not know the limiting degree distribution for infinite networks. On the other hand, because we are interested in understanding what processes could have given rise to realistic networks and given that realistic networks are never infinite, we are less interested in the limiting case. Besides, no real-world network is a *"*true power law". Finally, in the sentence you quote, we state that we *"*confirm that the scale-free distribution is unavoidable". We are referring here to previous work that showed that duplications give rise to scale-free distributions. We should have put a citation there, and we have done so now*.

"...the most "pure" scale-free networks actually emerge if nodes are added more often than edges". This is a very vague statement. How is the 'purity' of a scale-free distribution measured and how much more often does one need to add nodes than edges to get distributions of a certain 'purity'?

**Authors' response**: *We agree, this sentence could have been clearer. We now do not refer to the *"*purity" of a scale-free edge distribution any more: this term clearly is colloquial. Instead, we now write that *"*Networks that are scale-free over more decades actually emerge if nodes are added more often than edges". You are right, one could conceivably quantify this effect: we have left this for other users to test*.

"choosing a low *P*_*N*_, on the other hand, leads to the growth of networks with a Poissonian degree distribution". I don't see at all why the authors think that Figure 2B shows a Poissonian degree distribution. In fact, intuitively I would guess that to get a real Poisson distribution one probably needs a limit *P*_*E*_/*P*_*N *_→ ∞.

**Authors' response**: *We agree, this distribution is not exactly a Poisson distribution. As we mentioned earlier, very few natural processes lead to exact Poisson distributions. For example, the degree distribution of the C. elegans neuronal network is often referred to as *"*Poissonian", but in fact cannot be fit by a Poisson distribution. It is perhaps an abuse of language, but we often refer to a *"*Poisson-like" distribution as *"*Poissonian". Nevertheless, we have changed that sentence by replacing *"*Poissonian" with *"*Poisson-like"*.

"We conclude that the edge distribution can be controlled entirely...". It is of course clear that, as *P*_*N*_,*P*_*E*_, and *P*_*D *_are changed, one can get different degree distributions. But WHICH degree distributions one can get is really not clear. It is not clear to me if one can get true Poissonian or true power-law distributions in any limit. Clearly figure 3 suggests that the distributions one obtains might actually be quite complex mathematically (containing a minimum at some value of n that probability depends on the size to which the network is grown). What would really be helpful is some DERIVATION of what kind of networks are obtained for what kind of parameters in the limit of large network size.

**Authors' response**: *As mentioned earlier, our goal is not to derive a mathematical theory about the kind of edge distributions that can be generated using certain processes in the limit of infinite networks or ratios of probabilities that approach zero or infinity, but to provide a tool that allows users to test hypotheses about processes, and a means to create distributions that are qualitatively similar to what we can see in nature*.

The section on modularity I had no problems with although again the authors don't present a general theory for how modularity depends on all parameters, but rather just present a few illustrations in which one parameter is varied.

**Authors' response**: *Just like you, we would also very much appreciate a general theory that predicts how modularity depends on all parameters. But we understand that understanding how a single parameter determines the modularity using first principles is already a difficult undertaking. For example, we previously had a section in the manuscript that investigates the dependence of modularity on the gene duplication probability, but found that the interaction with the other probabilities painted a very complex picture that could not be covered by a subsection, and we instead relegated that discussion to a subsequent manuscript. In that manuscript, we will also present a mathematical analysis of the modularity as a function of a few parameters, but even that manuscript will fall short of your call for a theory that predicts the modularity of a network as a function of all parameters*.

page 9: "...the "small world" effect: the observation that many biological and technological networks have a short mean path between nodes (as compared to an equivalent randomized network)" I am not an expert but when I heard Duncan Watts speak about this about a decade ago the definition of the term 'small world' seemed to be that the mean path between nodes grows only LOGARITHMICALLY with the number of nodes in the network. This means that Erdosz-Renyi graphs are in the small-world class. The authors here seem to define small world as: even smaller mean path length than in an Erdosz-Renyi graph. This is a totally different concept.

**Authors' response**: *While the concept we use may not be exactly that advocated by Watts and Strogatz a decade ago, it is the most modern measure of *"*small-worldness" to date, and quantitative to boot. It is described in detail Ref*. [53].

Regarding equation (7), what happens when the number of edges is such that it is below the percolation threshold in the Erdosz-Renyi graph, i.e., so that the graph will fall into many small ones? How is the mean path length then defined and how is the ratio in equation (7) determined?

**Authors' response**: *We thank the referee for pointing out this missing piece of information. When calculating the mean shortest path for networks that fall into unconnected subnetworks, we calculate the mean of shortest paths in the subnetworks (weighted by the size of the network) as is usual. For the parameter regime that we investigated, you can convince yourself that we are far from the percolation threshold, so that most nodes are in the largest connected component anyway*.

"..which can be different from the clustering coefficient obtained by averaging the number of edges of the adjacent nodes." I didn't understand this remark. What precisely is being averaged?

**Authors' response**: *This should have read: *"*(...) averaging the number of edges between adjacent nodes". Thanks for pointing this out*.

page 10: "the ratio of these quantities can be used to measure small-world-ness". Again this seems confused to me. Small-world-ness in my understanding refers to the logarithmic scaling of mean distance with network size.

**Authors' response**: *Humphries and Guerney (Ref*. [53]) *have provided a more quantitative measure, which is the one we are using*.

Watts/Strogatz introduced a class of networks that combined the high clustering coefficient observed in many real world networks (and in lattices) with the logarithmically growing mean distance also observed in some real world networks (and in Erdosz-Renyi graphs). The point was that, starting from a lattice, you only need a small number of edges between randomly picked pairs of nodes, to change the scaling of mean distance from power-law (*N*^1/dimension^) to logarithmic in *N*. So a 'small world network' in the Watts/Strogatz sense is one that has high clustering AND logarithmically growing distance. It thus by definition requires 2 things to hold and cannot be quantified be easily quantified by a single number.

**Authors' response**: *The ratio that Humphries and Guerney define does both things, and manages to quantify small-worldness by a single number. They incidentally show this by applying their ratio to the network analyzed by Watts and Strogatz*.

The illustration on page 10 again shows just 1 particular example from which it is very hard to infer any general rules. For example, take this statement: "*L*_*g*_/*L*_random _actually increases for decreasing *q *as long as *P*_*N*_/*P*_*E *_< 2." How are we to understand this? Would this hold for different values of *q *and *PD *or for different size networks?

**Authors' response**: Figure 9*shows an average over 1,000 nodes, for a ratio of node to edge addition probabilities between 0.05 and 2, thus covering most of the interesting region, for two values of q that were representative (we have checked the whole range of q, but such a graph is not more instructive than the one we are presenting). It is true that different gene duplication probabilities will affect the graph. But the investigation of gene duplication on small-worldness and graph modularity is the subject of a different manuscript. The observation that L*_*g*_/*L*_random _*increases for decreasing q is noteworthy because smaller q results in edge randomization of the network, which at first glance would imply that the ratio should decrease. The reason it increases in the region mentioned is because edge randomization can also connect disconnected parts of the graphs, thus increasing L*_*g*_.

page 11: "We observe that the percolation phase transition only depends on the ratio *P*_*E*_/*P*_*N *_". As I explained above, the way the model is defined it seems the graph structure per definition should only depend on the ratio *P*_*E*_/*P*_*N *_when *p *= *q *= 1 and *P*_*D *_= 0.

**Authors' response**: *We agree, this sentence could be clearer. Indeed the transition depends on P*_*D*_, *as we show in *Fig. 12. *What we meant to say is that, given P*_*D*_, *the transition depends only on the ratio. We have made this clearer in the text*.

"allowing for node duplication does not affect.... but do not affect its emergence." This is not so obvious to me. Clearly duplications do not connect different components of the graph, i.e. it only grows them, but it seems to me that duplications do affect the distribution of sizes of the components and so I would expect *S *to depend on *P*_*D*_. On a related note, again it seems that the dependence on the final size of the network is not studied. This is kind of crucial here since one only really has a transition in the limit of infinite network size. Whether *S *is independent of *p *is also not clear to me.

**Authors' response**: *For the purpose of understanding the size of the largest component, node duplications act just like a node addition with concomitant edge addition. Thus, node duplication cannot affect the critical point, which is all we are saying. Also, there is no final size of the network, so we cannot study this (nor can anyone else)*.

Figure 10. Why on earth are networks only grown to size 100 in this figure? Surely one needs much bigger networks to talk about a transition. That is, one needs to show that the curves become independent of network size for large enough networks.

**Authors' response**: *We learned from experience that networks grown to 100 nodes are not qualitatively different from those grown to 100,000 or even tens of millions of nodes when it comes to determining the relative size of the largest connected component. At P*_*E*_/*P*_*N *_= 1, *the size of the largest component is very close to 0.6 for networks of 100 nodes, which is almost indistinguishable from the relative size measured by Callaway et al*. [43]*for a 17 million node network grown with the same parameters*.

'Biological relevance' section. I have a few comments here also. First, I don't see why there is any need to bin the edge distribution.

**Authors' response**: *It turns out that conventional curve-fit routines are terrible in fitting the long tail of a power law, because the counting errors there are so large. Often, counts are included with a single observation at that size, and no observations for any of the adjacent sizes. Binning the data into bins that contain at least a fixed number of observations is an effective method to remove this problem. In Ref*. [61]*one of us shows that the curve that is fit through data binned using this method is for all intents and purposes identical to that which is generated by statistics so good that binning is not necessary. In other words, this binning method produces a distribution that is indistinguishable from the theoretical distribution creating the sample. In order to calculate a *"*goodness score" for our simulated network, we need to compare it to the data, which doesn't have any entries in many of the edge numbers. Binning allows you to compare meaningfully*.

Given enough simulations it should be easy to calculate the likelihood to obtain the observed degree distribution using the model at a given parameter setting. Alternatively, one could calculate something like a Kolmogorov-Smirnov statistic to assess how close the degree distributions of the grown networks are to the observed one. This brings me to my second point. Even for the 'best' grown networks I bet that a Kolmogorov-Smirnov test would reject the hypothesis that the degree distributions are the same. That is, although the authors' model allows one to grow 'similar' degree distributions, it seems pretty clear to me that the model does actually NOT allow one to reproduce the observed degree distribution within statistical noise.

**Authors' response**: *Based on your suggestion, we have performed a statistical test to assess the closeness of the degree distributions. The appropriate test here is to determine whether two independent samples both are drawn from the same underlying probability distribution (as the biological datum is technically not a probability distribution, we should not be using the Kolmogorov-Smirnov test). We used a non-parametric test (the Wilcoxon rank-sum test), and show that we cannot reject the hypothesis that the biological datum and the simulation are samples taken from the same underlying probability distribution (see new text). In other words, this is a bet that you would have lost. In fairness, we note that we decided to remove data from the yeast edge distribution for which the number of edges exceeds 120 (removing exactly eight proteins out of 3,306 from the data set). We removed those because it makes no sense to fit data this sparse, which also does not appear to follow the degree distribution of the remaining 3,298 proteins. We note this removal in the manuscript*.

Second, why is only the degree distribution looked at? What about the clustering coefficients and distribution of distances between nodes? Wouldn't one want to see if the grown networks also reasonably approximate these statistics of the real world networks?

**Authors' response**: *It was not the object of this comparison to show that the simulated networks are identical to biological ones. Surely we can test whether other statistics are also well-approximated, and if they don't then we can refine our parameter search in such a manner that we perhaps satisfy any additional constraint. The point is that nothing would be sufficient to prove that the parameters we find are actually those that drove the biological process, so pushing the similarity of the networks further is a somewhat futile undertaking. The title of the manuscript emphasizes degree distributions, so we focused on that character*.

page 15: "Another standard control... while keeping the total number of edges and nodes the same." Really? Won't this in general change the number of edges and in fact lead to a graph in which half of all possible edges exist on average?

**Authors' response**: *For the parameter setting that we discuss there, the number of edges does not change on average, as an edge is removed as often as one is added. The process instantiates a random walk in *"*edge number space" where the mean stays constant*.

As a general remark about the conclusions, the claims that the authors make about what they have done are much more grandiose than what has really been done in my opinion. As already pointed out, nowhere is a general theoretical analysis presented of what kind of combinations of topological features can and cannot be produced within this growth model. To give just one example: to what extent can degree distributions, clustering coefficients, and distances be tuned independently? Over what range?

**Authors' response**: *We suppose that the size of claims is in the eye of the beholder, but several times in this reply we have emphasized that our claims are really very modest, and that instead you have several times imputed claims to us that we either do not make, or do not intend to make. Furthermore, we have repeatedly pointed out that some of your expectations of what it is that we should be presenting in this manuscript are either unrealistic or downright impossible to realize. We readily agree that a reader should not walk away from this manuscript believing that we can grow any desired network with just a few parameters, and we have included language in the introduction that makes that caveat, while toning down any passage that could be misunderstood as claiming that the model is *"*universal"*.

"These networks, however, are entirely devoid of any function". How can one know this? It all depends on the function. There are some circumstances where a network that is perfectly optimized for a given function can be automatically generated using just the kind of stochastic growth process that the authors are using.

**Authors' response**: *We responded to this misunderstanding earlier: all we state is that we show that a particular feature (*"*form") can emerge without selection for function, so that we can reject the hypothesis that function is necessary for the evolution of form. It would be silly to claim that the resulting form can never be useful for any function (and we do not make this claim)*.

### Reviewer 2: Teresa Przytycka, NIH/NLM/NCBI (nominated by Claus Wilke)

Hintze and Adami propose a general model to grow networks with diverse degree distributions. Their model uses a small number of intuitive parameters - conditional probability of node/edge addition/deletion and node duplication. The authors propose that by appropriately adjusting these parameters it is possible to construct a network with an arbitrary degree distribution. Indeed, they demonstrated that the method provides means for iterative generation of a large number of different types of networks. The authors also demonstrated (experimentally) that modifying in a continuous way leads to a "phase transition" in the properties of generated network. Another interesting property of their network growing algorithm is that it facilities growing modular networks (utilizing the functional modularity measure introduced by the authors). These are indeed interesting observations. Missing from the presentation is some kind of graphical presentation of the parameters landscape-which parameters lead to which type of distribution. Admittedly, not everything can be summarized in one figure, and not all combination of parameters tested, but some kind of summary figure or table would be very helpful.

**Authors' response**: *This is a very helpful suggestion. We have followed your advice and introduced a new Figure *(Fig. 5) *that summarizes how some key parameters affect the degree distribution. It is only a qualitative figure, but certainly paints a much more intuitive picture*.

To be precise, the authors didn't prove that their method allows for growing networks with arbitrary degree distribution. Rather they showed that networks with some degree distributions can be generated. Even for the network types discussed in the paper, the argument is mostly informal-based on visual inspection of the degree distribution of generated graph. It would be more correct to state, that they postulate that this method allows for growing networks with broad range of degree distributions and provide computational simulations to support this postulate. I doubt that truly arbitrary degree distribution can be achieved by this approach.

**Authors' response**: *We are grateful for your remarks. We are now aware that the structure of the paper does not adequately reflect our intentions. While it is true that we can generate arbitrary degree distribution if we engage the help of the adjacency matrix, it is also true that arbitrary degree distributions cannot be obtained using the five main independent parameters. We have modified the abstract and included a new paragraph in the introduction that makes these restrictions clear*.

The concept of anti-modularity is very unintuitive. How does it differ from simply not being modular and what are examples of anti-modular network, if any? Can the authors provide an example of a network that is not modular and is not anti-modular?

**Authors' response**: *Anti-modular networks are networks where nodes of the same kind preferentially do not attach to each other. Typical examples are bi-partite networks, for example dating networks where the majority of edges are between nodes classified as opposite gender. But there are also such examples in biology, e.g., gene regulatory networks where some nodes are transcription factors and others are DNA binding sites*.

The authors also demonstrated that that they are able to adjust the parameters of the model to generate networks approximating degree distribution of selected biological network. While this is interesting, it immediately raises the question whether the networks (simulated and real) are similar in a more broad sense. Clearly, vertex degree distribution is just one measure of a network property. If the proposed network growing algorithm truly mimics evolutionary scenario, similarities of other properties of the network, such as diameter, distribution of small sub-networks (such as the graphlets proposed by Przulj), connectivity, etc. should be also be observed. It would be important to check if this is indeed the case.

**Authors' response**: *This comment echoes the one made above by Erik, and we sympathize with the feeling inasmuch as if a whole manuscript were devoted to the issue, then we certainly would want to follow exactly the path proposed by you and Erik. In fact, we have another manuscript where we are investigating the distribution of motifs in real and simulated C. elegans neuronal connection graphs. But such an analysis would explode the boundaries of this manuscript: in the section *"*Biological relevance" we simply set out to test whether the degree distribution of some well-known networks can be approximated, as this is after all a claim that we are making. We have changed the wording in that paragraph to make sure that our ambition is not misunderstood*.

### Reviewer 3: Leonid Mirny (Massachussetts Institute of Technology)

This reviewer provided no comments for publication.
